# Evaluation of Anticancer Activity of Novel Sulfanyl-Substituted Hydrazone Compounds in Hepatocellular Carcinoma: In Vitro, In Silico, and In Ovo Studies

**DOI:** 10.1021/acsomega.5c13125

**Published:** 2026-03-05

**Authors:** Hatice BAŞPINAR KÜÇÜK, Tülay YILDIZ, Yaren ARASAN, Buse Meriç AÇAR, Duygu KURTOĞLU, Aslı KUTLU, Remzi Okan AKAR, Sinem KILIÇ, Demet GÜL ERYILMAZ, Engin ULUKAYA

**Affiliations:** † Faculty of Engineering, Department of Chemistry, Organic Chemistry Division, Istanbul University-Cerrahpasa, Istanbul 34320, Turkey; ‡ Molecular Cancer Research Center (ISUMKAM), Istinye University, Istanbul 34396, Turkey; § Institute of Graduate Education, Molecular Oncology Program, Istinye University, Istanbul 34396, Turkey; ⊥ Faculty of Engineering and Natural Science, Department of Molecular Biology and Genetics, Istinye University, Istanbul 34396, Turkey; # Faculty of Medical School, Department of Clinical Biochemistry, Istinye University, Istanbul 34396, Turkey

## Abstract

Hepatocellular carcinoma is one of the hardest-to-treat cancer types, although some recent developments have been made. Therefore, the discovery of new treatment options is still desperately desired. In this study, some novel sulfanyl-substituted hydrazone compounds **2a–2h** were synthesized and characterized using spectroscopic techniques (^1^H NMR, ^13^C NMR, IR, and HRMS). Then, hydrazone compounds were tested on four different cancer types (lung, hepatocellular, breast, and colon carcinoma) and one nonmalignant cell line. Among these hydrazone compounds and tested cancer types, compound **2c** resulted in a satisfactory antigrowth effect against hepatocellular carcinoma, the HepG2 cell line. Further, in ovo antitumor and antiangiogenic assays were also performed together with the in silico calculations employed by target predictions of compound **2c** by a tool called Way2Drug and then followed by binding affinity calculations to those targets by AutoDock Vina. The range of binding scores of compound **2c** was calculated between −4 and −8.6 kcal/mol for those targets that were suggested to be involved in a local protein–protein interaction clustering on base excision repair and DNA topological change. It has been found that compound **2c** might deserve further attention (e.g., animal studies for proof-of-concept) as a novel anticancer compound for the treatment of liver cancer.

## Graphical abstract

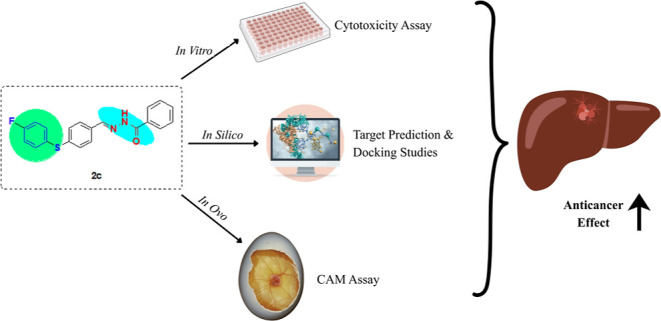

## 1 Introduction

Liver cancer poses a major public health challenge on a global scale, standing as the sixth most frequently diagnosed malignancy and the third highest cause of cancer-related mortality worldwide.^1^ Hepatocellular carcinoma (HCC) represents the dominant form of primary liver cancer, accounting for around 75% to 85% of all cases.^2–5^ In general, conventional therapeutic strategies that focus on targeting a single molecular site or signaling pathway often fail to provide long-term and effective tumor control as cancer cells are capable of activating compensatory survival mechanisms, reprogramming their metabolism, and employing escape pathways under therapeutic pressure. These adaptive responses frequently result in major clinical challenges, including reduced drug efficacy, recurrence, and the emergence of multidrug resistance. Therefore, novel anticancer compounds are still required.^6^

Hydrazide–hydrazones represent a class of organic compounds characterized by the presence of both a hydrazide and a hydrazone moiety, typically connected through an azomethine linkage (–CO–NH–N=CH–). These compounds have gained considerable attention due to their wide range of biological activities, particularly their promising anticancer potential.^7–10^ The anticancer properties of hydrazide–hydrazone derivatives have been previously investigated, revealing that many such molecules exhibit notable cytotoxic activity against various cancer cell lines.^11–15^ In comparison to simple hydrazides, hydrazide–hydrazones seem to have superior pharmacological profiles. They undergo hydrolysis in both in vitro and in vivo environments, resulting in less toxic but pharmacologically active metabolites. This unique biotransformation pathway contributes to their reduced systemic toxicity and enhanced therapeutic potential.^12,16,17^ Moreover, the chemical masking of the –NH_2_ group within the hydrazide linker increases their applicability in prodrug design, making them more attractive candidates for the development of targeted anticancer therapies. Certain pharmaceutical agents containing hydrazide–hydrazone or hydrazone moieties with varying substitutions are already integrated into the structures of agents currently utilized in cancer therapy as exemplified by 5-fluorouracil, bisantrene, nifuroxazide, mitoguazone, and zorubucin.^18–21^

Recently, there has been increasing interest in incorporating electron-withdrawing and electron-donating groups into various compounds to modify their chemical and pharmacological properties.^22,23^ Furthermore, it has been observed that the addition of thioether linkers to bioactive molecules generally favorably affects the selectivity of these agents against cancer cells compared to normal cells.^24^ The biological significance of the hydrazone scaffold,^25–28^ combined with our interest in physiologically active chemicals, prompted us to develop a new class of hydrazone cores with diverse substituted sulfanyl aromatic groups to explore their impact on biological activity. In our previous study, a series of novel *N*-acyl hydrazone derivatives from 2-arenoxybenzaldehyde were synthesized, resulting in the identification of lead compounds which displayed promising antitumor activity.^29,30^ Thus, this study is a continuation of our work in the field of hydrazone compounds. In the newly synthesized compounds, the main backbone was formed with benzoyl or furoyl hydrazone groups. Moreover, we adopt a sulfanyl-substituted phenyl ring with various electron-donating or -withdrawing groups to the hydrazone backbone. This design strategy aimed to establish preliminary structure–activity relationships (SARs) by correlating electronic and structural variations with cytotoxic responses in cancer cells. Then, the newly synthesized compounds **2a**–**2h** were tested on various cancer cell lines to evaluate their biocompatibility and anticancer activity, respectively. The biological evaluation showed that compound **2c** exhibited a dose-dependent cytotoxic/antigrowth effect against HepG2 hepatocellular carcinoma cells. Relatively lower concentrations such as 3.12 μM caused a moderate decrease in growth, while concentrations of 6.25 μM and above resulted in a nearly 40–50% reduction in viability. These findings suggest that compound **2c** has anticancer properties, as proved by in vitro and in ovo assays (antitumoral and antiangiogenic effect) together with the in silico calculations, thereby warranting further investigation for the treatment of liver cancer.

## 2 Results and Discussion

### 2.1 Chemistry

To obtain the proposed derivatives, the synthesis was initiated with the Ullmann coupling reaction to produce substituted 4-phenylsulfanyl-benzaldehydes (**1a–d)**.^29,30^ Subsequently, these aldehydes (**1a–d**) were reacted with benzoic acid hydrazide or furan-2-carboxylic acid hydrazide in ethanol under reflux conditions, yielding hydrazones with sulfanyl-substituted aromatic groups (**2a–2h**) in high yields (Scheme 1).

**Scheme 1:**
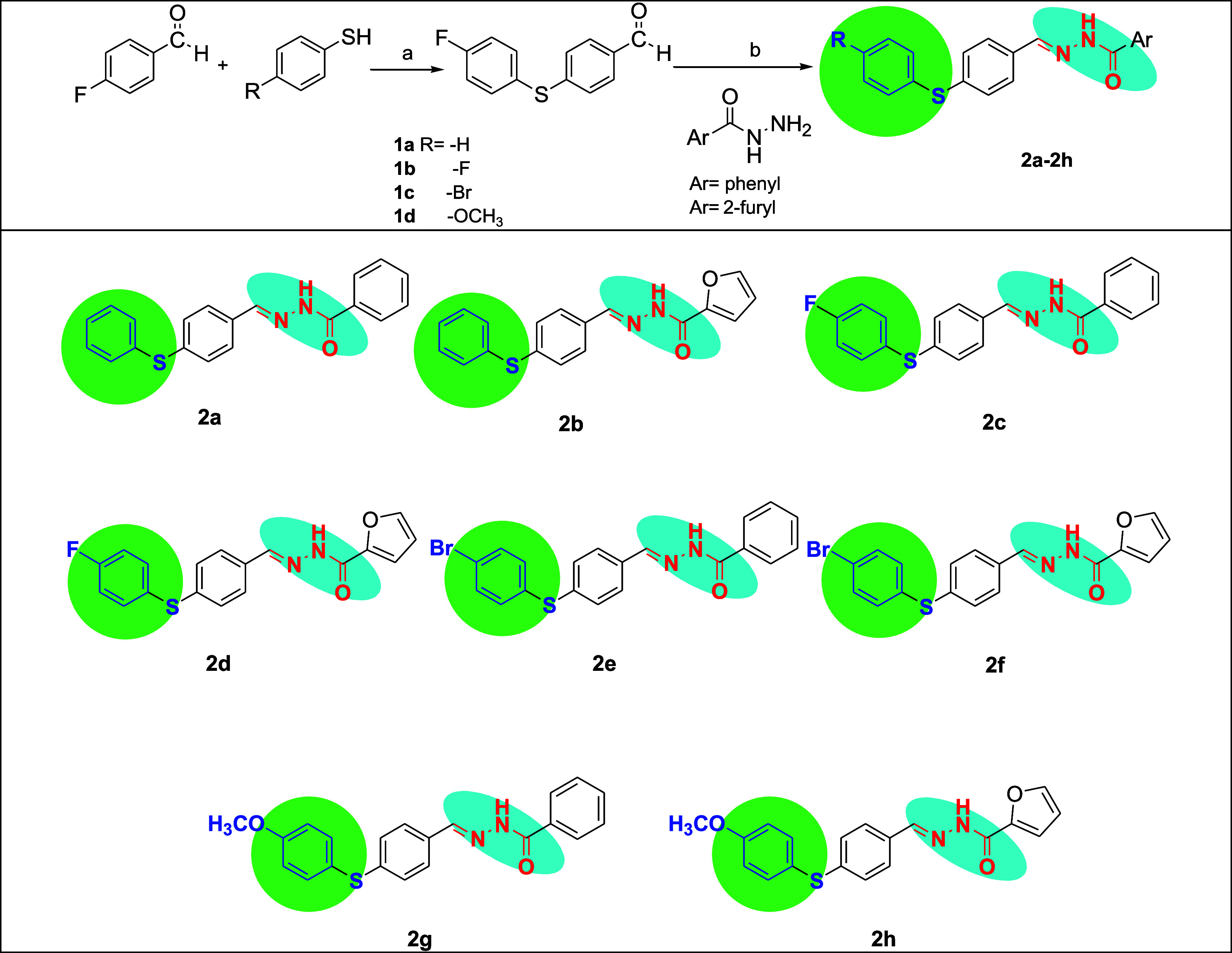
Synthesis of Target Compounds **2a–2h** ^a^

a Reagents and conditions: (a) K_2_CO_3_, DMF, 155 °C, 2 h. (b) Absolute ethanol, reflux, 2–5 h.

In this work, different substituents at sulfanyl-substituted aromatic groups (such as F, Br, and OCH_3_) and hydrazide derivatives (benzoic acid hydrazide or furan-2-carboxylic acid hydrazide) were utilized to examine the structure–activity relationships (SARs) of the title compounds as well as the influence of the types of groups on anticancer activities. The synthesized compounds were isolated by filtration and subsequently recrystallized from ethanol, affording good to excellent yields (84%–94%). Structural characterization was performed using ^1^H NMR, ^13^C NMR, FT-IR, HRMS, and melting point analysis. The FT-IR spectra of compounds **2a–2h** exhibited characteristic NH stretching bands within the range of 3294–3178 cm^–1^. Additionally, in the IR spectra of compounds **2a–2h**, absorption bands observed in the 1666–1643 cm^–1^ region were assigned to C=O stretching vibrations. In the ^1^H NMR spectra of compounds **2a**–**2h**, the N–H and azomethine (CH=N) protons were observed as singlets at 11.82–11.94 ppm (1H, s) and 8.39–8.49 ppm (1H, s), respectively. In addition, the ^13^C NMR spectral data also confirmed the presence of imine carbon (NH–N=C) in these compounds. The HRMS data were also in agreement with the assigned structures. In support of the Experimental Section, the Supporting Information (SI) includes the ^1^H and ^13^C NMR and HRMS spectra of all novel compounds.

### 2.2 Biological Activity

The initial viability/antigrowth screening was conducted at a concentration of 10 μM for each compound on a panel of cancer cell lines, including MDA-MB-231 (triple-negative breast cancer), A549 (lung adenocarcinoma), HCT116 (colorectal carcinoma), and HepG2 (hepatocellular carcinoma), as well as the nonmalignant human bronchial epithelial cell line BEAS-2B. Among the tested compounds, compound **2c** exhibited notable antigrowth activity, particularly against the HepG2 cell line (Figure 1). In further viability and growth rate analyses, six doses were used: 100 μM, 50 μM, 25 μM, 12.5 μM, 6.25 μM, and 3.12 μM. It was found that cell proliferation was inhibited in a dose-dependent manner on the HepG2 cell line (Figure 2A). After 48 h of treatment, a marked reduction in cell viability was observed, particularly at concentrations of 6.25 μM and above. The IC_50_ dose was determined to be 23 μM, and the GI_50_ dose was determined to be 6.25 μM. These findings indicate that compound **2c** possesses a moderate antigrowth potential. Therefore, compound **2c** emerges as a promising pharmacological candidate for further investigation in hepatocellular carcinoma.

**Figure 1:**
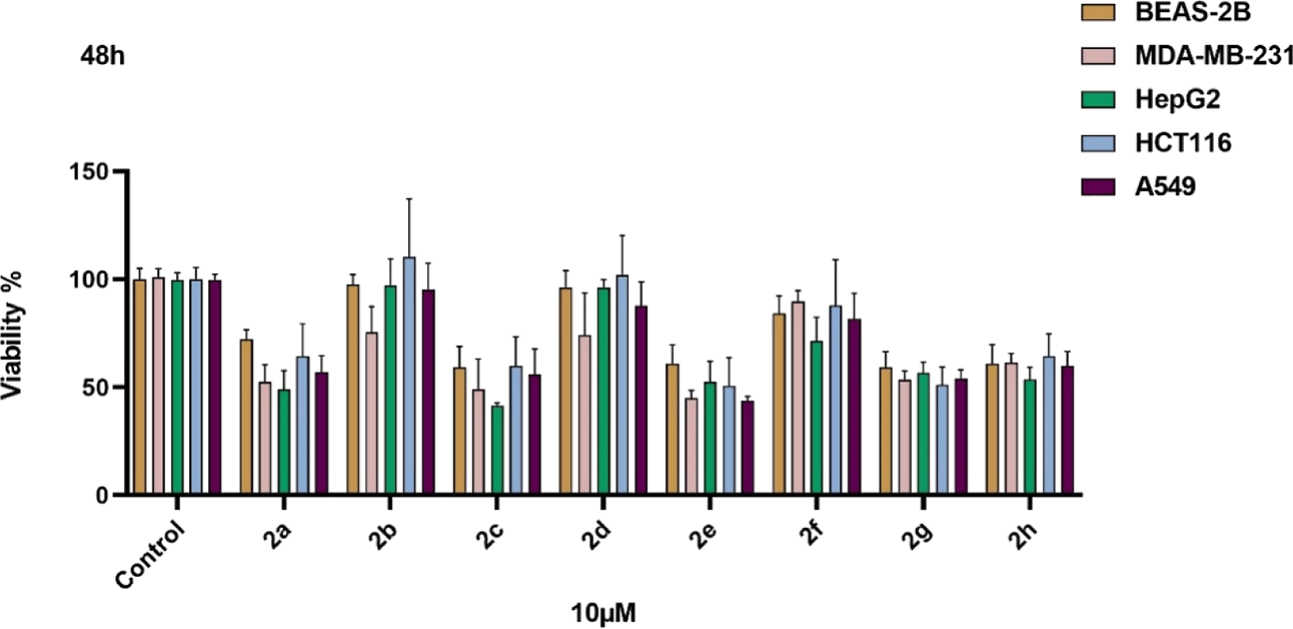
Initial screening of hydrazone compounds **2a**–**2h** at 10 μM dose on four different cancer cell lines and a normal human bronchial epithelium cell line.

**Figure 2:**
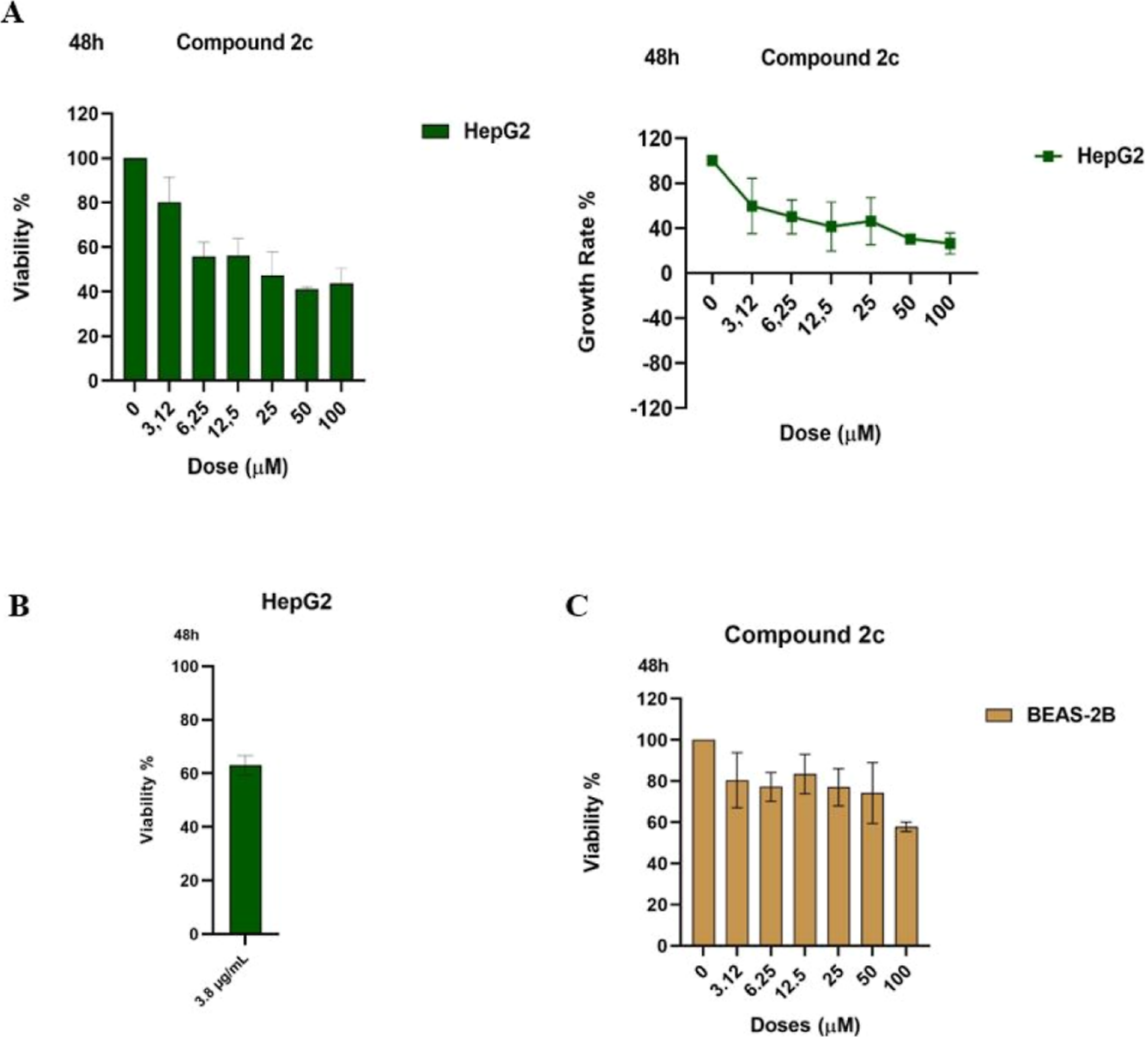
A) Further screening of compound **2c** on the HepG2 hepatocellular carcinoma cell line at six different doses. (B) Treatment with a clinically relevant plasma peak dose of cisplatin. (C) Further screening of compound **2c** in the BEAS-2B nonmalignant human bronchial epithelium cell line.

As a reference chemotherapeutic agent, cisplatin was included due to its widespread clinical use in the treatment of various solid tumors, including hepatocellular carcinoma.^31,32^ Plasma peak concentration of cisplatin can reach peak levels in the low microgram per milliliter range, with reported *C*_max_ values of approximately 2–4.8 μg/mL depending on the dose and infusion protocol.^33,34^ In this study, cisplatin was applied at a concentration of 3.8 μg/mL, which is within the range of the clinically relevant peak plasma concentration observed in patients receiving standard cisplatin chemotherapy (Figure 2B).

In order to evaluate the selectivity of compound **2c**, cell viability analysis was also performed on the nonmalignant human bronchial epithelial cell line BEAS-2B (Figure 2c). Compound **2c** exerted a lower cytotoxic effect on BEAS-2B cells compared to HepG2 cells, indicating a favorable selectivity profile toward malignant cells. Notably, cell viability in BEAS-2B cells was partly preserved at concentrations that significantly inhibited the proliferation of HepG2 cells. Based on IC_50_ values obtained from dose–response analyses, the selectivity index (Supporting Information) of compound **2c** was calculated to be 5.4, further supporting its specific antigrowth effect against HepG2 cancer cells over nonmalignant cells.

### 2.3 Structure–Activity Relationship (SAR) Analysis

The hydrazone derivatives **2a–2h** were evaluated for antiproliferative activity against four human cancer cell lines (MDA-MB-231, HepG2, HCT116, and A549) and the normal human bronchial epithelial cell line BEAS-2B. All compounds share a common structural framework comprising a benzoyl or furoyl hydrazone core linked to a sulfanyl-substituted phenyl ring, allowing for meaningful structure–activity relationship (SAR) analysis based on structural variations.

Among the cancer cell lines, MDA-MB-231 cells showed moderate sensitivity to several derivatives. In contrast, HCT116 and A549 cells were comparatively less sensitive, suggesting that molecular targets or uptake mechanisms relevant to this scaffold may be less prominent in these cell types. Against the HepG2 cell line, the tested derivatives showed different levels of antiproliferative activity. The fluoro-substituted benzoyl derivative **2c** was the most potent, followed by the highly active benzoyl analogues **2a** and **2e**. The methoxy-substituted compounds **2g** and **2h** showed moderate activity, whereas derivatives **2f**, **2d**, and **2b** were weakly active or inactive. In HepG2 cells, compound **2c** exhibited the lowest viability among the tested derivatives, identifying it as the most active compound at the screening concentration. Structurally, **2c** combines a benzoyl hydrazone moiety with a fluoro-substituted thiophenyl ring, suggesting that this substitution pattern is especially favorable for activity in liver cancer cells.

Comparison of benzoyl derivatives (**2a**, **2c**, **2e**, and **2g**) with their corresponding furoyl analogues (**2b**, **2d**, **2f**, and **2h**) reveals that benzoyl substitution consistently enhances activity in HepG2 cells. In each matched pair, benzoyl analogues produced lower cell viability than their furoyl counterparts, highlighting the importance of an extended aromatic acyl group for activity. The benzoyl group is more lipophilic than the furoyl moiety, as the furan oxygen introduces additional polarity that may reduce membrane permeability. Enhanced lipophilicity in the benzoyl series may facilitate improved cellular uptake in HepG2 cells (Figure 3).

**Figure 3:**
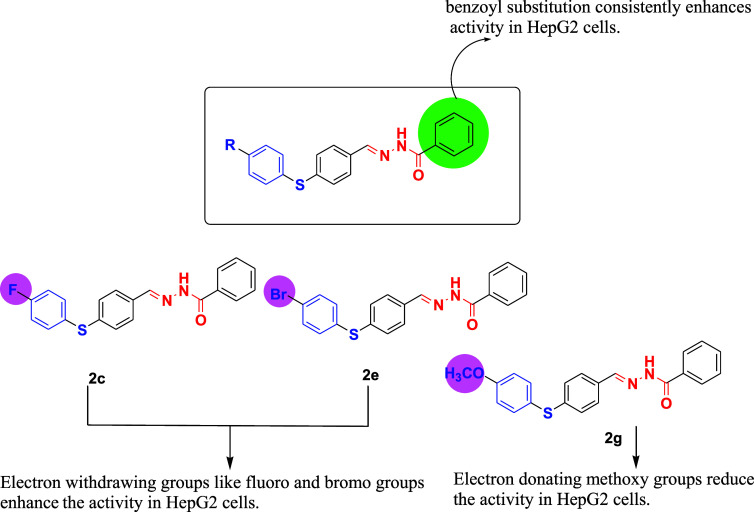
Structure–activity relationship of hydrazone compounds **2c**, **2e**, and **2g**.

Substituent effects on the thiophenyl ring further revealed a HepG2-specific preference. The fluoro-substituted derivative **2c** outperformed both the unsubstituted compounds (**2a**, **2b**) and the brominated analogues (**2e**, **2f**). Although bromine substitution improved activity relative to that of unsubstituted analogues, it did not surpass that of the fluoro derivative. While both fluorine and bromine are halogens, they differ substantially in their steric demand. Fluorine exerts strong electronic effects with a minimal steric effect. In contrast, bromine is considerably bulkier. The highest activity of **2c** therefore suggests that the relevant binding environment in HepG2 cells may be sterically constrained, favoring small substituents such as fluorine while disfavoring bulkier groups like bromine. This shows that a moderately electron-withdrawing group combined with a minimum steric volume is optimal for HepG2 activity. Methoxy-substituted compounds (**2g** and **2h**) displayed notably reduced activity in HepG2 cells. This indicates that electron-donating substitution on the thiophenyl ring is unfavorable for HepG2 inhibition. The loss of activity associated with the methoxy group highlights the importance of electronic effects: methoxy is a strong electron-donating group, whereas fluoro and bromo substituents act as electron-withdrawing groups via inductive effects. These results suggest that an electron-deficient thiophenyl ring is beneficial for activity.

In summary, the SAR analysis reveals that HepG2 cells display a distinct sensitivity profile, favoring compounds that combine a benzoyl hydrazone core with a fluoro-substituted thiophenyl ring.

### 2.4 Target Prediction

The compound **2c**, for which the two-dimensional chemical structure was available, was first subjected to target prediction using the Way2Drug platform. This initial screening step enabled the identification of potential molecular targets with high probability scores, thereby providing a rational basis for subsequent computational validation. The relationship between compound **2c** and potential targets was determined using the CLC-Pred (Cell Line Cytotoxicity Predictor) 2.0 web service.^35^ The prediction results are expressed as two probability values: Pa (probability of activity) and Pi (probability of inactivity). The Pa value indicates the likelihood that a compound might exhibit a specific biological activity, whereas the Pi value reflects the probability that the compound might be inactive for that activity. Both values range from 0 to 1, and Pa + Pi < 1, as they are calculated independently.^35^ According to the PASS software interpretation guidelines, a higher Pa value relative to Pi suggests a greater probability that the predicted biological activity can be experimentally confirmed. The cutoff value for Pa values at this prediction was set to 0.4 (Table 1). Subsequently, the expression levels (from normal tissues and liver hepatocellular carcinoma—LIHC tissues) of the potential targets included in this scope were compared using GEPIA2 for LIHC^36^ (Figure 4).

**Table 1:** Possible Compound **2c** Targets Predicted by Way2Drug

Pa	Pi	mechanism of action	UniProt code	protein name
0,876	0,004	trace amine-associated receptor 1 antagonist	Q96RJ0	TAAR1
0,678	0,06	ATP-dependent DNA helicase Q1 inhibitor	P46063	RECQL
0,648	0,06	lysosomal alpha-glucosidase inhibitor	P10253	GAA
0,631	0,012	DNA ligase 1 inhibitor	P18858	LIG1
0,622	0,063	DNA polymerase beta inhibitor	P06746	POLB
0,533	0,046	tyrosyl-DNA phosphodiesterase 1 inhibitor	Q9NUW8	TDP1
0,49	0,002	histone chaperone ASF1A inhibitor	Q9Y294	ASF1A
0,475	0,09	pyruvate kinase PKM inhibitor	P14618	PKM
0,465	0,037	phospholipase A2 inhibitor	P04054	PLA2G1B
0,442	0,024	sentrin-specific protease 6 inhibitor	Q9GZR1	SENP6
0,439	0,088	aldehyde dehydrogenase 1A1 inhibitor	P00352	ALDH1A1
0,434	0,094	methyl-CpG-binding protein 2 inhibitor	P51608	MECP2
0,433	0,011	26S proteasome non-ATPase regulatory subunit 14 inhibitor	O00487	PSMD14
0,431	0,099	neuropeptide S receptor antagonist	Q6W5P4	NPSR1
0,427	0,019	serine/threonine-protein kinase WNK2 inhibitor	Q9Y3S1	WNK2
0,41	0,036	sentrin-specific protease 7 inhibitor	Q9BQF6	SENP7
0,407	0,005	tyrosine-protein phosphatase nonreceptor type 5 inhibitor	P54829	PTPN5
0,407	0,027	protein mono-ADP-ribosyltransferase PARP16 inhibitor	Q8N5Y8	PARP16
0,406	0,049	polyunsaturated fatty acid lipoxygenase ALOX12 inhibitor	P18054	ALOX12

**Figure 4:**
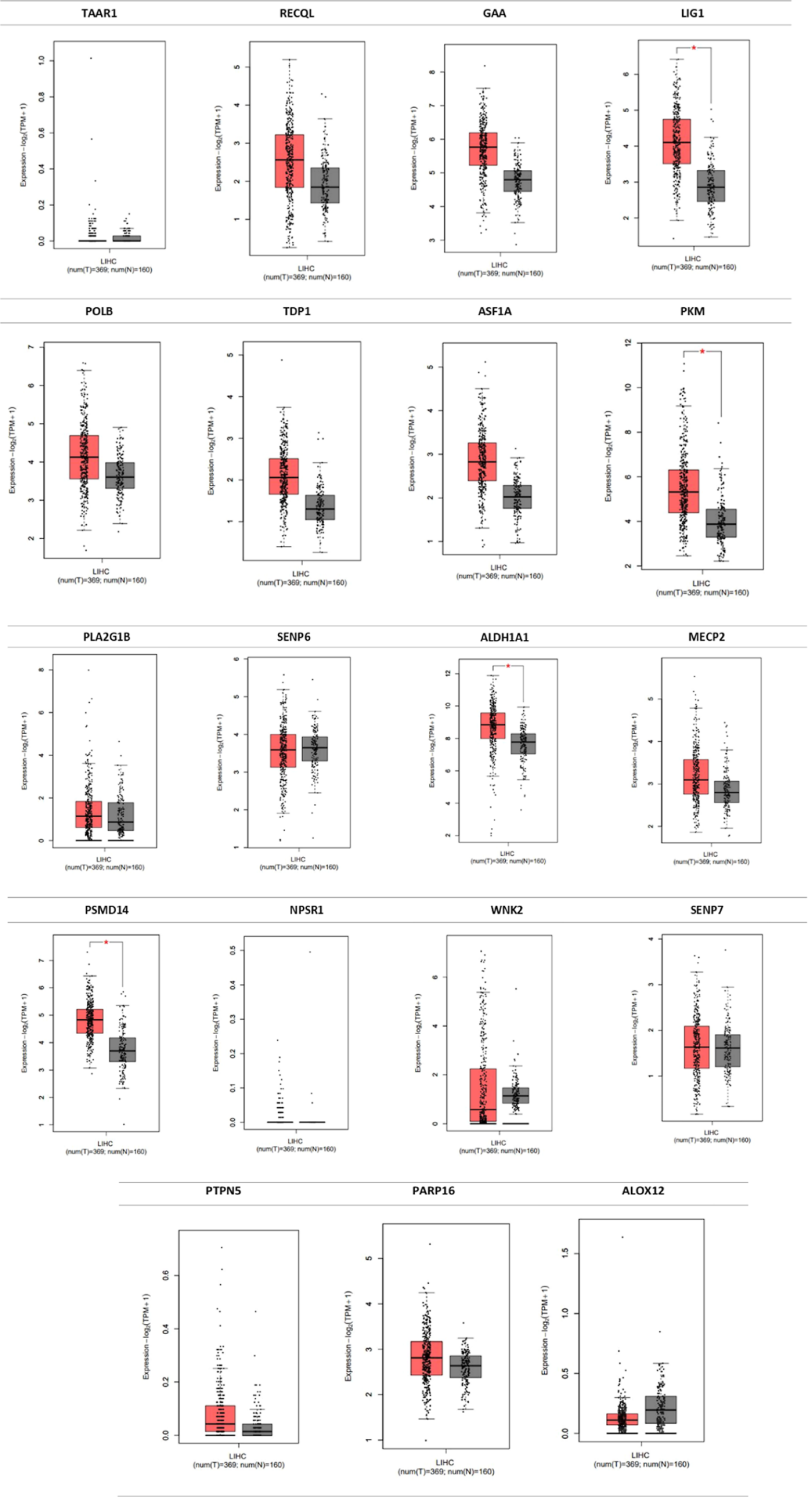
Expression levels of compound **2c**’s potential targets at LIHC (from GEPIA2).

### 2.5 Molecular Docking Studies

After the refinement of target pools of compound **2c**, docking studies were conducted with these predicted compound **2c** targets, whose gene expression levels are presented in Figure 4. Since all these targets have been coming from the output of the Way2Drug tool by giving the SMILES formula of compound **2c** as an input, the possible binding potential of compound **2c** together with the binding pose to those molecular targets has been explored more by the molecular docking approach. Only PSMD14 given in Figure 4 is excluded from the target pool for further docking evaluations due to the lack of its qualified full structure in the Protein Data Bank.

Prior to docking calculations, these target molecules are also analyzed in the STRING-db tool to check any possible protein–protein interactions among them. As displayed in Figure S25, we report the protein–protein interactions between LIG1, TDP1, RECQL, and POLB proteins. These protein–protein interactions are suggesting a local clustering by covering LIG1, TDP1, and POLB proteins on base excision repair and DNA topological change with a 0.0018 FDR value. The strongest one is reported between POLB and LIG1 with a 0.996 score, and it is followed by TDP1-LIG1 interactions with a 0.963 score if the default settings are used to run. In terms of the interactions that we observed, the DNA repair mechanism (GO term: 0006281) and catalytic activity acting on DNA (GO term: 0140097) are highlighted as the biological process and molecular functions with 0.0191 and 0.0098 FDR values, respectively.

During the docking calculations of compound **2c** to those targets, the grid box includes the active site of each receptor that is retrieved from the literature findings. Here, we performed docking calculations only to predict the possible binding affinity of compound **2c** to those targets coming from Way2Drug. The pose and score details of compound **2c** are provided in Table S1 with pose-specific docking scores together with RMSD values. As presented in Table 2, even these docking scores are mostly in the range of −4––8.6 kcal/mol in terms of compound **2c**’s affinities to them. In addition to that, we also calculate the *K*_d_ values as an inhibition constant for compound **2c** to each predicted targets, and the results are presented in Table 2 (see Table 3).

**Table 2:** Docking Scores and Coordinating Residues (* Indicates the Catalytic and/or Coordinating Residues of the Corresponding Receptor)

protein complex	docking score	*K*_d_ value (μM)	coordinating residues
RecQ1-chain A	–7.6 kcal/mol	2.68	Arg93*, Glu96*, Lys119*, Ser120*, Gln147, and Ile378
RecQ1-chain B	–7.9 kcal/mol	1.61	Arg93*, Lys119*, Ser120*, Gln147, Asp219* and Gln 220*
GAA	–7.4 kcal/mol	3.76	Asp404, Trp481*, Arg600* Asp616*, and His674*
LIG 1	–8.6 kcal/mol	0.49	Glu566*, Lys568*, Tyr569, Gln570, Arg573*, Glu621 and Leu743
PolB-active site	–6.9 kcal/mol	8.76	Ser104, Asp190*, Arg254 and Tyr271
PolB-5′-deoxyribose phosphate (5′-dRP)	–4.0 kcal/mol	1169.428	Lys35*, Tyr39 and Lys87
TDP1	–6.1 kcal/mol	33.77	Tyr204*, Cys205, His263, Ser400, Ser459, Pro461*, Asn516, Ser518, Ser536 and Glu538
ASF1A	–6.0 kcal/mol	39.99	Glu51, Asp54*, Val92*, and Val95
PKM2	–7.2 kcal/mol	5.27	His29, Met30, Asn318, Arg319, Asp354 and Glu397
ALDH1A1	–8.3 kcal/mol	0.82	Asn121, Asn170, His293*, Cys302*, and Cys302
MeCP2 + DNA complex	–8.4 kcal/mol	0.69	no hit within protein structure; bind to DNA
MeCP2 w/o DNA	–6.7 kcal/mol	12.27	Lys119, Tyr120, Asp121, and Lys135

**Table 3:** In Ovo Antiangiogenic Scoring System

score	antiangiogenic effect	after treatment effect on CAM
0	inactive	no change
0.5	weak	no capillary-free area
1	strong	small capillary-free area or area with significantly reduced density of capillaries.
2	very strong	capillary-free area around the pellet at least double the size of the pellet

$Average\;score=\frac{\left(score\;2\right)\#egg\times 2+\left(score1\right)\#egg}{total\;\#egg\;scored}$

RecQ1 (ATP-dependent DNA helicase Q1) plays essential roles in DNA replication, repair, and the resolution of DNA secondary structures, thereby preventing replication stress and maintaining genomic stability.^37^ Elevated RecQ1 expression has been reported in several cancers, including hepatocellular carcinoma (HCC), where it promotes tumor cell proliferation, invasion, and survival under genotoxic stress.^38–40^ In the literature, the active site of RECQ1 is suggested as Arg93, Gln96, Gly118, Lys119, Ser120, and Asp219 ^41^. All these listed residues are compromised in the grid box (52 Å × 40 Å × 70 Å for chain A and 60 Å × 54 Å × 72 Å for chain B) required for RecQ1 (PDB ID: 2 V1X) and compound **2c** docking.

The docking calculations of compound **2c** to the 3D structure of RecQ1 (2 V1X) were run for both two dimeric assemblies (chain A and chain B) (Figure S26). Here, we report that the docking scores of compound **2c** to RecQ1_A and RecQ1_B were −7.6 and −7.9 kcal/mol, respectively. The contact analysis has suggested that RecQ1-A and RecQ1-B chains have different preferences to coordinate compound **2c** such that Arg93, Glu96, Lys119, Ser120, Gln147, and Ile378 are coordinating compound **2c** in RecQ1-A and Arg93, Lys119, Ser120, Gln147, Asp219, and Gln 220. By using the docking scores of compound **2c** to RecQ1 chain A and chain B, we also calculated *K*_d_ values of compound **2c** as 2.68 μM and 1.61 μM, respectively. According to the findings in the literature, the strong association between depletion of RecQ1 and anticancer effects has been reported, and this makes RecQ1 an attractive target for anticancer.^42,43^ Upon the effective binding of compound **2c** to the ATP-coordinating region of RecQ1, it most probably happened that the ATP-dependent helicase activity of RecQ1 has been disrupted well, and therefore, the unwinding of DNA cannot take place. This actually indicates the existence of genomic instability that implies the existence of cell death for tumor cells.

Acid α-glucosidase (GAA) is a lysosomal hydrolase that catalyzes glycogen degradation to glucose. Beyond its established role in Pompe disease, recent studies highlight that lysosomal glycogen breakdown (glycophagy) contributes to metabolic flexibility in cancer cells, allowing them to adapt to nutrient stress and then to sustain proliferation.^44^ Structural and biochemical characterization of human GAA has defined key residues within its binding pocket of the catalytic domain, including Leu405, Trp481, Trp516, Met519, Phe525, Arg600, Asp616, Phe649, Leu650, His674, Ser676, Leu677, and Leu678 (Malik et al., 2024). To calculate the binding ability of compound **2c** to the GAA protein structure (PDB ID: 5KZX), all these listed residues were included into the definition of the grid box with 60 Å × 58 Å ×70 Å dimensions. As a result, we calculated a −7.4 kcal/mol docking score between the GAA protein structure and compound **2c**, and it has a *K*_d_ value of 3.76 μM. The coordination of compound **2c** within GAA was coordinated by Asp404, Trp481, Arg600, Asp616, and His674 (Figure S27). Among these coordinating residues, only Asp404 is not part of the active site in the GAA protein complex but has a role in keeping compound **2c** within the protein complex.

As displayed in Figure 4, there is an increase of GAA expression level in tumor cells compared to control ones, even though it is not significant with p-val <0.05. This particular increase in the amount of GAA expression may indicate the accelerated lysosomal glycogen degradation in tumor cells. If there exists a targeted inhibition of GAA, it would be associated with the lysosomal dysfunction due to the accumulation of undigested glycogen in lysosomes. Hence, this process would end up with the promotion of cell death by autophagy as a result of disrupted autophagic flux. In the literature, there are several findings about the benefician of the GAA inhibition mechanism to overcome chemoresistance, developed by gemcitabine treatment and associated with the elevated GAA expression level, together with leading to an increase in the response of the cell death mechanism.^45,46^ Especially for the cancer therapies in which gemcitabine has been offered as a first-line therapeutic option, the alternative strategies to inhibit GAA would be a great option to trigger the lysosomal–autophagy axis.

LIG1 (DNA ligase 1) plays a critical role in DNA replication and repair. Due to its critical role in several DNA repair mechanisms, such as the requirement for it to connect newly synthesized DNA fragments and thereby determine the efficiency of DNA synthesis, numerous studies have investigated LIG1’s possible roles in various cancer types. For example, it can be used as a biomarker to indicate the response rate to immunotherapy and the presence of chemoresistance.^47–50^ LIG1 inhibition strategies would be a great alternative to contribute to the cancer treatments.^50^

Within the 3D structure of LIG1 (PDB ID: 1X9N), there is an ATP-binding pocket within the adenylation domain, and there are several key residues involved in ATP coordination and catalysis: Glu566, Lys568, Arg573, Glu621, Phe635, Phe660, Glu720, Met723, and Trp742 ^51^. Lys568 forms the covalent interaction with the AMP intermediate during the first step of the ligation reaction, and herein Arg573 and Glu621 contact with the OH group of AMP ribose by contributing to ATP specificity. Within this architecture, the role of Glu720 is to coordinate the metal ion (Mg2+) closely located to the pyrophosphate linkage, and Trp742 assists to exclude noncognate nucleotides such as GTP through steric and electronic complementarity with the adenine base. Even though Phe635 and Fhe660 are not directly contacted with ATP, they have a role in stabilizing the local interactions within the active site of LIG1 and hence create a favorable environment for DNA nicking.^51^ This well-established architecture within LIG1 has been highly preserved to ensure its proper functioning. Through docking of compound **2c** to the ATP-coordinating site of LIG1 with a 56 Å × 50 Å ×40 Å grid size, we calculate a −8.6 kcal/mol docking score with a 0.49 μM *K*_d_ value by the involvement of the following residues: Glu566, Lys568, Tyr569, Gln570, Arg573, Glu621, and Leu743 (Figure S28).

POLB (DNA polymerase β) is a key enzyme in base excision repair (BER). Overexpression or mutationally altered POLB has been associated with genomic instability and tumorigenesis in various cancers, and POLB perturbation in experiments has been shown to affect tumor cell proliferation and chemosensitivity.^52,53^ Its expression level is also used as a prognostic marker for platinum-based chemo-treatments.^54^ Specifically in HCC, it has been revealed that the overexpression of PolB in HCC has been associated with the poor prognosis^55^ as being linked to the circadian clock mechanism.

To check the possible binding conformations of compound **2c** to POLB, we select the 3D structure of POLB with PDB ID: 1BPX, representing the catalytically active state following nucleotide-induced subdomain closure.^56^ Within the 3D architecture of PolB, there are two functionally distinct regions: (1) 5′-deoxyribose phosphate (5′-dRP) binding region and (2) active site. Within the 5′-dRP binding region, Lys35, Lys68, and Lys72 play a critical role for recognition and excision of the 5′-deoxyribose phosphate (5′-dRP) intermediate during base excision repair (BER). For the active site of PolB located in the C-terminal, there are Arg149, Arg183, Gly189, Asp190, Asp192, Asp256, Asp276, Arg258, and Phe272 residues involved in the coordination of incoming dNTP, catalytic Mg^2+^ ions, and the primer terminus to facilitate nucleotidyl transfer.^57^ To run docking calculations, we have performed two approaches: (1) to keep the deoxyribose phosphate (5′-dRP) structure within PolB for ensuring the proper binding of compound **2c** by setting our grid box into its active site with a 52 Å × 48 Å × 40 Å size and (2) to check whether there is any competitive binding between compound **2c** and deoxyribose phosphate (5′-dRP) by setting our grid box into the 5′-dRP’s binding site with a 40 Å × 40 Å × 40 Å size.

Our docking calculations have suggested that compound **2c** is coordinated by Ser104, Asp190, Arg254, and Tyr271 in the active site of POLB with a −6.9 kcal/mol docking score and an 8.76 μM *K*_d_ value and in the 5′-deoxyribose phosphate (5′-dRP) binding site of PolB with a −4.0 kcal/mol docking score and a 1169.428 μM *K*_d_ value (Figure S29). The targeted inhibition mechanism of POLB may provide an advantage for proper use of synergetic therapies, including DNA-damaging agents, for enhancement of cytotoxicity at the cellular level, and for overcoming chemoresistance.

TDP1 (tyrosyl-DNA phosphodiesterase 1) is an enzyme involved in DNA repair. The elevated TDP1 activity can contribute to resistance against TOP1 poisons (e.g., camptothecins).^58,59^ Pharmacologic or genetic TDP1 inhibition potentiates TOP1-targeted chemotherapy by increasing persistent DNA damage, replication stress, and apoptotic signaling in tumor cells, making TDP1 a promising combinatorial target.^60^

Within the 3D structure of TDP1 (PDB ID: 7UFZ) that includes the catalytically active N-terminal truncation, the active site coordinating residues are listed as Tyr204, His263, Lys265, Asn283, Gln294, Ser399, Ser459, Pro461, His493, Lys495, Ser514, and Asn516, and these residues are covered by setting the docking grid box with a 48 Å × 54 Å × 54 Å size. Among these listed residues, His263, Lys265, His493, and Lys495 correspond to the canonical HKD motifs characteristic of the phospholipase D (PLD) superfamily and are directly implicated in nucleophilic attack and transition-state stabilization. Here, Asn283, Gln294, Asn516, and Ser514 contribute well to the H-bond networking that modulates the electrostatic interactions within its active site. Moreover, Tyr204, Ser399, Ser459, and Pro461 help shape the substrate-binding cleft that accommodates the tyrosine–DNA phosphodiester linkage.^61^ Our docking results have suggested that compound **2c** binds to TDP1 with a −6.1 kcal/mol binding score and a 33.77 μM *K*_d_ value, by the involvement of the following residues: Tyr204, Cys205, His263, Ser400, Ser459, Pro461, Asn516, Ser518, Ser536, and Glu538 (Figure S30).

ASF1A (Anti-Silencing Function 1A Histone Chaperone) has a role in chromatin dynamics and DNA replication and repair.^62^ Its dysregulation alters the chromatin dynamics and hence promotes the uncontrolled proliferation.^63^ Overexpression of ASF1A has been observed to facilitate epithelial–mesenchymal transition (EMT), invasion, and poor prognosis in many cancers including HCC,^64^ and ASF1A-mediated chromatin changes can influence oncogenic transcriptional programs.^65,66^ Therefore, targeting ASF1A seems to be a novel strategy to limit the invasion and stemness capacity by improving response to treatment.^65,67^

To run docking calculations, the 3D structure of ASF1A is taken from PDB (PDB ID: 6F0F) by including N-terminal domains between 1 and 156 residues.^68^ The grid box for docking calculations was selected within the C-terminal helix (α3) of histone H3 (residues 122–135), in which Glu49, Asp54, Val92, Val94, and Tyr112 residues are located. These regions are very critical and are stated as an interface to provide control over ASF1A–H3/H4 interactions. Here, the surrounding charged residues Asp54 and Glu49, together with Tyr122, contributed to the reshaping of electrostatic interactions to stabilize the binding interface for ASF1A–H3/H4. Our docking calculations have suggested that compound **2c** binds to the ASF1A receptor with a −6.0 kcal/mol binding score and a 39.99 μM *K*_d_ value, with the involvement of the following coordinating residues: Glu51, Asp54, Val92, and Val95 residues (Figure S31).

There are two forms of pyruvate kinase (PKM): PKM1, which is constitutively active and supports oxidative phosphorylation rather than glycolysis, and PKM2, which is frequently upregulated in tumors and supports aerobic glycolysis, as well as nonmetabolic functions such as nuclear signaling and transcriptional coactivation, which promote proliferation, survival, and therapy resistance.^69^ PKM2 is frequently overexpressed in tumors, supporting aerobic glycolysis and nonmetabolic functions such as nuclear signaling and transcriptional coactivation, which promote proliferation, survival, and therapy resistance.

To run docking calculations, we retrieved the 3D structure of the PKM2 protein with PDB ID: 4G1N^70^. This conformation covers the canonical active site of PKM2 that is required for the glycolytic isozyme. Here, the grid structure for docking calculation with a 60 Å × 52 Å × 50 Å size is composed of Phe26, His29, Met30, Lys311, Asn350, Gly355, Ile389, Tyr390, Gln393, Leu394, and Gly397. The role of these residues is to form the binding interface for phosphoenolpyruvate (PEP), which binds to A- and C-domains of PKM2. Here, Lys311 participates in the binding and orientation of phosphate groups of PEP, while Tyr390 and Gln393 contribute to the hydrophobic and polar environment that stabilizes the enolpyruvate transition state.^71^ Our docking calculations have suggested that compound **2c** binds to the PKM2 protein complex with a −7.2 kcal/mol docking score and a 5.7 μM *K*_d_ value. The following residues are involved in the coordination of compound **2c** to PKM2: His29, Met30, Asn318, Arg319, Asp354, and Glu397 (Figure S32).

ALDH1A1 (aldehyde dehydrogenase 1A1) is considered a functional marker of cancer stem-like cell populations in multiple tumor types, contributing to the detoxification of reactive aldehydes, resistance to chemotherapeutics, and self-renewal capacity.^72,73^ High ALDH1A1 activity correlates with tumorigenicity, metastasis, chemoresistance, and a worse clinical outcome in several malignancies, including HCC.^74,75^ Therefore, the well-targeted inhibition of ALDH1A1 leads to depletion or impairment in the self-renewal capacity of cancer stem cells to reduce tumor initiation, metastasis, and recurrence.

To run docking calculations, the 3D structure of ALDH1A1 is retrieved from the Protein Data Bank (PDB ID: 4WJ9).^76^ Within the 3D architecture of ALDH1A1, the catalytic residues are listed as Trp178, His293, and Cys 302 ^77^ ^,^ ^78^. Therefore, these critical residues are included in the grid box with a 52 Å × 54 Å × 64 Å size for running docking calculations. We report that the docking score of compound **2c** to ALDH1A1 is −8.3 kcal/mol and 0.82 μM *K*_d_ value, and here Asn121, Asn170, His293, Cys302, and Cys302 residues are involved in the coordination of compound **2c** (Figure S33).

MeCP2 (methyl-CpG binding protein 2) has been linked to oncogenic transcriptional programs.^79^ Experimental studies have shown that MeCP2 can promote proliferation, suppress apoptosis, and facilitate metastasis in cancers such as hepatocellular carcinoma and breast cancer.^80,81^ The expression level of MeCP2 in HCC is also used as a predictive marker to discriminate cirrhosis- or noncirrhosis-based HCC,^82^ and its elevated expression level in HCC indicates poor survival.^83^ Specifically for HCC, there are several critical findings about the inhibition of MeCP2 such that siRNA-induced MeCP2 inhibition has resulted in the reduced proliferation of HepG2 cells by decreasing the cell activity and division.^84^

To run docking calculations, the crystal structure of the MeCP2 protein including the methyl binding domain has been retrieved (PDB ID: 6OGJ).^85^ Within the protein architecture, there are critical residues having a role in methylated DNA recognition such as Arg111, Arg133, Ser134, and Lys135. The detailed structural analysis of MeCP2 has suggested that Arg111 and Arg133 serve as “arginine fingers” to form H-bonds required for the binding of both methylated CpG (mCG) and unmethylated GTG-containing DNA motifs to the major groove. Here, Ser134 and Lys135 are closely located to the DNA backbone to confer the H-bonding network together and electrostatic interactions that are required for effective binding of DNA to MeCP2. Therefore, we included these critical residues to run docking calculations.

Specifically for the MeCP2 protein, we run two different conditions to calculate the docking score of compound **2c**: (1) inclusion of DNA with MeCP2 with grid size 106 × 84 × 78 Å and (2) exclusion of DNA within MeCP2 with grid size 92 × 80 × 80 Å. For case 1, we report a −8.4 kcal/mol docking score of compound **2c** with a 0.69 μM *K*_d_ value to MeCP2, but there are no coordinating residues within the receptor. The reason is that compound **2c** has a higher affinity to bind DNA, and therefore, there are no closely located residues to coordinate the binding of compound **2c** (Figure S34­(A)). Even though we have made several attempts to organize the grid box within the receptor (MeCP2-DNA complex) to offer a suitable binding surface for compound **2c**, it always prefers to bind DNA molecules within the complex. But for case 2, we report a −6.7 kcal/mol docking score of compound **2c** with a 12.27 μM *K*_d_ value to the receptor in which it has just been composed of MeCP2. For MeCP2 conformation without DNA binding, compound **2c** is coordinated by Lys119, Tyr120, Asp121, and Lys135 (Figure S34­(B)).

Lastly, it is also worth stating that even though PSMD14 was predicted as a target of compound **2c** and its expression level was significantly different between tumor and control tissue samples, its docking calculation has resulted in a positive docking score toward PSMD14, and it indicates no proper binding of compound **2c**. Therefore, it is not discussed as a possible target of compound **2c** in HepG2.

Based on the docking calculations of compound **2c** toward its predicted targets, we conclude that LIG1, MeCP2, and ALDH1A1 are reported as the most promising targets of compound **2c** when the docking scores and *K*_d_ values are taken into consideration. It is important to note here that the calculated *K*_d_ values of compound **2c** for these targets are below the experimentally identified viability dose of 6.25 μM. Moreover, those targets have a role in the DNA repair mechanism, and this common point highlights the potential role of compound **2c** to target the DNA repair mechanism from varied perspectives. As previously suggested, LIG1 has a critical role in several DNA repair mechanisms, and targeting its inhibition would be a great alternative to offering new treatment approaches. For MeCP2, it is stated specifically for HCC that its elevated expression level is a signature of poor survival, and again, its inhibition would have a potential for a positive treatment response. Also, for ALDH1A1, it would be very promising to perform well-targeted inhibition of ALDH1A1 due to leading the depletion or impairment in the self-renewal capacity of cancer stem cells to reduce tumor initiation, metastasis, and recurrence. Also, it is important to discuss RecQ1 as the next strong target of compound **2c**, followed by the top three ones due to the binding score and *K*_d_ value. Specifically, for RecQ1 expression in HCC, it has been suggested that its high expression level promotes cell proliferation, invasion, and survival under genotoxic stress, and therefore, its targeted inhibition might work well to provide a better treatment profile in HCC.

### 2.6 Assessment of Antitumoral and Antiangiogenic Effects by an In Ovo CAM Assay

Quantitative evaluation of tumor growth on the chick chorioallantoic membrane (CAM) assay was performed using three complementary analytical approaches: direct area measurement via ImageJ, ellipsoid volume estimation, and spherical volume calculation. All analyses were based on scale-normalized images captured at identical magnification. As shown in Figure 5A, digital imaging demonstrated that the control tumors maintained a similar size between baseline (Tz) and 48 h, indicating minimal growth within this period. In contrast, tumors treated with compound **2c** exhibited a visibly smaller size at 48 h compared with their baseline images, suggesting an inhibitory effect on tumor expansion. Quantitative assessment across all three measurement approaches confirmed this observation (Figure 5B). The mean tumor volume in the control group corresponded to approximately 85% of the baseline (Tz) volume (set as 100%), whereas the compound **2c**-treated group showed a mean tumor volume of approximately 65%, reflecting a 20% reduction relative to the control. These results consistently demonstrate that compound **2c** significantly attenuates tumor growth on the CAM within 48 h of treatment at the IC_50_ dose. Literature studies have also shown that hydrazone treatments at comparable doses yielded similar results in in ovo experiments.^86,87^

**Figure 5:**
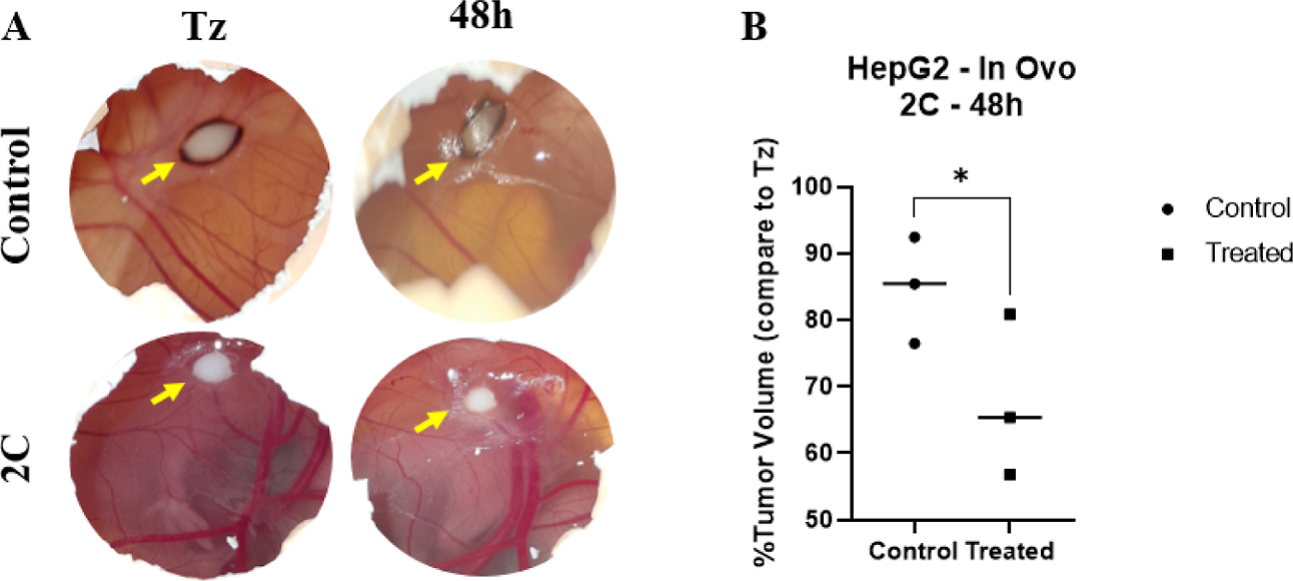
A) HepG2 cancer cells and Matrigel graft on the CAM on day 14. (B) Tumor volume change comparison graph.

The agreement among the three analytical methods reinforces the reliability of the finding, indicating that the effect is not an artifact of the measurement technique. Given the rapid and measurable reduction in tumor volume, compound **2c** likely exerts antiproliferative and/or antiangiogenic activity, consistent with previously reported outcomes observed in ovo CAM xenograft models of hepatocellular carcinoma and other tumor types.^88,89^ To evaluate the antiangiogenic potential of the test compound, a dose-dependent CAM assay was performed using three concentrations (1000, 100, and 10 μM). The vascular alterations on the CAM surface were assessed at 24 and 48 h following topical compound application. Antiangiogenic response was quantified using a scoring system. At the 1000 μM dose, a pronounced inhibition of angiogenesis was observed (Figure 6). The mean score at 24 h was around 1.5, reflecting a reduction in the total vessel area. Interestingly, the antiangiogenic effect slightly decreased by 48 h, yielding a mean score around 1.0. At 100 μM, the compound exerted a moderate but measurable inhibitory effect. The mean scores were around 1.0 at 24 h and 0.5 at 48 h, indicating a sustained yet diminishing antiangiogenic response over time. At the lowest tested dose (10 μM), minimal inhibition was detected. The 24 h mean score remained below 0.5, while no detectable antiangiogenic activity (score = 0) was evident at 48 h, suggesting that this concentration is insufficient to impair CAM vascularization.

**Figure 6:**
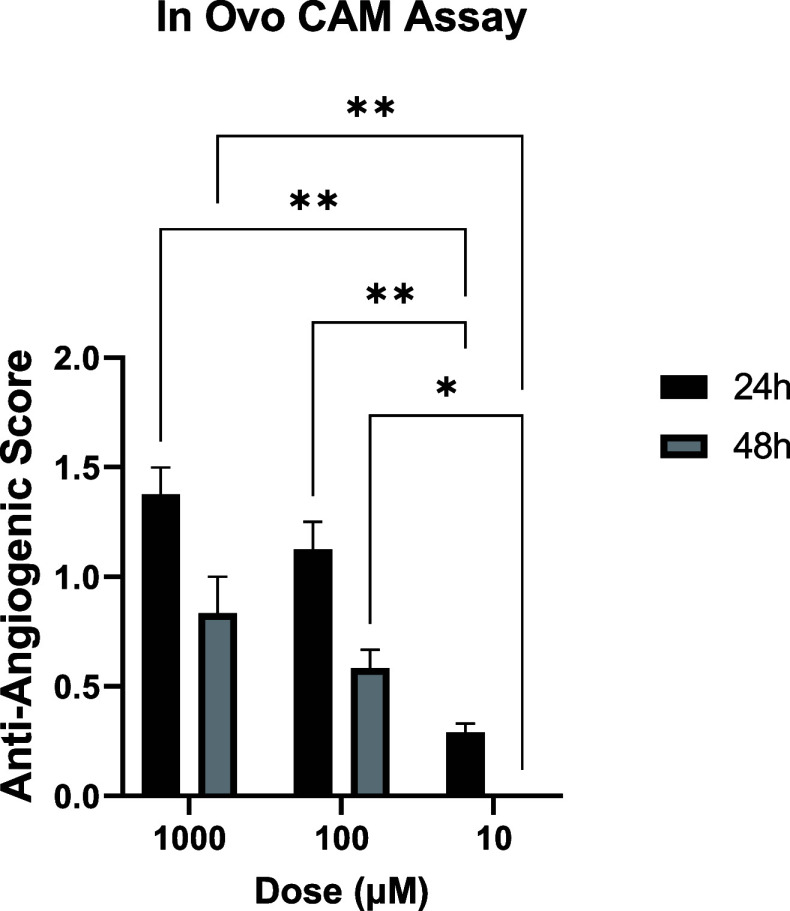
Antiangiogenic score change comparison graph.

Overall, the results demonstrate a clear dose-dependent antiangiogenic activity, with significant inhibition occurring at 1000 μM and detectable but weaker effects at 100 μM. These findings are consistent with the compound’s cytotoxic and antiproliferative properties observed in vitro and further support its potential as a candidate with antivascular activity.

## 3 Conclusion

In this study, a new series of sulfanyl-substituted hydrazone compounds **2a–2h** were synthesized via Ullmann-type coupling, followed by condensation reactions. Among the synthesized compounds, **2c** exhibited a significant concentration-dependent cytotoxic effect against the HepG2 hepatocellular carcinoma cell line and reduced cell viability at a dose of 6.25 μM. Additionally, in ovo antitumor and antiangiogenic effect analyses yielded promising results toward its being an anticancer compound. Taken in silico analyses into consideration, these findings highlight the potential of compound **2c** as a possible inhibitor against LIG1, MeCP2, and ALDH1A1 in HCC. Together with all these experimental results and in silico calculations, compound **2c** can be viewed as a promising candidate. Future studies will focus on expanding the compound library, investigating the mechanistic basis of cytotoxic activity, and conducting in vivo animal evaluations. Particular emphasis will be placed on investigating the lead compound potential of these compounds to develop more effective and targeted cancer therapies.

## 4 Experimental Section

### 4.1 Chemistry

All of the starting materials were commercially available reagents and used without further purification. Solvents for chromatography were of technical grade and were distilled prior to use. Melting points were recorded with a Buchi melting point B-540 and uncorrected. ^1^H and ^13^C NMR spectra were recorded at 500 MHz for ^1^H and at 125 MHz for ^13^C using Me_4_Si as the internal standard in DMSO with a Bruker Avance NEO 500 MHz NMR spectrometer. Coupling constants were given in hertz (Hz). HRMS spectra were recorded on a Thermo Scientific Q Exactive hybrid quadrupole-orbitrap MS instrument. IR spectra were recorded on an Agilent Cary 630 FTIR spectrometer. All reactions were monitored by thin-layer chromatography (TLC) using silica-gel plates (silica gel 60 F254 0.25 mm).

#### 4.1.1 General Experimental Procedure of 4-Phenylsulfanyl-benzaldehydes (**1a–d**)

To a solution of DMF (10 mL) containing 4-fluorobenzaldehyde (5.0 mmol) and thiophenol (5.0 mmol) was added K_2_CO_3_ (5.0 mmol), and the reaction mixture was stirred for 2 h at 155 °C under a nitrogen atmosphere. It was cooled to room temperature, and after the usual workup and concentration, the product was purified over silica gel.

#### 4.1.2 General Experimental Procedure of Hydrazones (**2a–2h**)

To a solution of appropriate hydrazide (benzoic acid hydrazide or furan-2-carboxylic acid hydrazide) (2.0 mmol) in absolute ethanol, a stirred solution of substituted 4-phenylsulfanyl-benzaldehydes **1a–d** (2.0 mmol) was added. The solution was refluxed for 2–3 h. The solid product formed was collected by filtration and recrystallized with ethanol.

##### 4.1.2.1 Benzoic Acid (4-Phenylsulfanyl-benzylidene)-hydrazide (**2a**)

White powder, mp 179–180 °C, Yield: 84%. IR (cm^–1^) ν 3178 (NH), 2989 (CH, aryl), 1647 (C=O), 1543 (C=N), 1435 (NH), 1346 (C–N), 1288 (Ar–S), 748, 690 (CH, aryl). ^1^H NMR (500 MHz, DMSO) δ: 11.91 (s, 1H), 8.47 (s, 1H), 7.95 (d, *J* = 7.5 Hz, 2H), 7.73 (d, *J* = 8.0 Hz, 2H), 7.62–7.60 (m, 1H), 7.55 (t, *J* = 7.4 Hz, 2H), 7.46–7.39 (m, 5H), 7.34 (d, *J* = 8.0 Hz, 2H). ^13^C NMR (126 MHz, DMSO) δ: 163.60, 147.46, 138.53, 133.87, 133.71, 133.37, 132.47, 132.24, 130.27, 130.02, 128.95, 128.67, 128.46, 128.10. HRMS (ESI) (*m*/*z*): [M + H]^+^: calculated for C_20_H_16_N_2_OS: 333.1062, found: 333.1050.

##### 4.1.2.2 Furan-2-carboxylic Acid (4-Phenylsulfanyl-benzylidene)-hydrazide (**2b**)

White powder, mp 155–156 °C, Yield: 87%. IR (cm^–1^) ν 3228­(NH), 3066 (CH, aryl), 1651 (C=O), 1546 (C=N), 1469 (NH), 1354 (C–N), 1296 (Ar–S), 806, 756 (CH, aryl). ^1^H NMR (500 MHz, DMSO) δ: 11.88 (s, 1H), 8.44 (s, 1H), 7.95 (s, 1H), 7.68 (d, *J* = 8.3 Hz, 2H), 7.45–7.43 (m, 5H), 7.40–7.37 (m, 1H), 7.31 (d, *J* = 8.2 Hz, 2H), 6.70 (s, 1H). ^13^C NMR (126 MHz, DMSO) δ: 154.65, 147.52, 147.08, 146.33, 138.53, 133.71, 133.28, 132.44, 130.26, 130.02, 128.66, 128.44, 115.48, 112.57. HRMS (ESI) (*m*/*z*): [M + H]^+^: calculated for C_18_H_15_N_2_O_2_S: 323.0854, found: 323.0844.

##### 4.1.2.3 Benzoic Acid [4-(4-Fluoro-phenylsulfanyl)-benzylidene]-hydrazide (**2c**)

White powder, mp 192–193.5 °C, Yield: 90%. IR (cm^–1^) ν 3178 (NH), 3051 (CH, aryl), 1643 (C=O), 1558 (C=N), 1462 (NH), 1354 (C–N), 1280 (Ar–S), 759, 694 (CH, aryl). ^1^H NMR (500 MHz, DMSO) δ: 11.87 (s, 1H), 8.43 (s, 1H), 7.92 (d, *J* = 7.5 Hz, 2H), 7.70 (d, *J* = 8.1 Hz, 2H), 7.61–7.58 (m, 1H), 7.55–7.53 (m, 4H), 7.31 (t, *J* = 8.7 Hz, 2H), 7.27 (d, *J* = 8.1 Hz, 2H). ^13^C NMR (126 MHz, DMSO) δ: 163.71, 163.57 (d, ^1^ *J*_CF_ = 228.7 Hz), 161.75 (d, ^1^ *J*_CF_ = 228.7 Hz), 147.45, 139.19, 135.76 (d, ^3^ *J*_CF_ = 8.5 Hz), 135.70 (d, ^3^ *J*_CF_ = 8.5 Hz), 133.86, 133.15, 132.24, 129.20, 128.95, 128.45, 128.09, 117.54 (d, ^2^ *J*_CF_ = 22.1 Hz), 117.37 (d, ^2^ *J*_CF_ = 22.1 Hz). HRMS (ESI) (*m*/*z*): [M + H]^+^: calculated for C_20_H_16_N_2_OFS: 351.0967, found: 351.0955.

##### 4.1.2.4 Furan-2-carboxylic Acid [4-(4-Fluoro-phenylsulfanyl)-benzylidene]-hydrazide (**2d**)

White powder, mp 149.5–150.5 °C, Yield: 94%. IR (cm^–1^) ν 3194 (NH), 3059 (CH, aryl), 1651 (C=O), 1562 (C=N), 1465 (NH), 1346 (C–N), 1296 (Ar–S), 748, 671 (CH, aryl). ^1^H NMR (500 MHz, DMSO) δ: 11.86 (s, 1H), 8.42 (s, 1H), 7.94 (s, 1H), 7.67 (d, *J* = 8.3 Hz, 2H), 7.54–7.51 (m, 2H), 7.30 (t, *J* = 8.7 Hz, 3H), 7.26 (d, *J* = 8.2 Hz, 2H), 6.70 (s, 1H). ^13^C NMR (126 MHz, DMSO) δ: 163.70, 161.74, 154.65, 147.52, 147.07, 146.33, 139.19, 135.72 (d, ^2^ *J*_CF_ = 8.4 Hz), 135.66 (d, ^2^ *J*_CF_ = 8.4 Hz), 133.05, 129.20, 128.71, 128.43, 117.53 (d, ^2^ *J*_CF_ = 22.1 Hz), 117.35 (d, ^2^ *J*_CF_ = 22.1 Hz), 115.46, 112.96. HRMS (ESI) (*m*/*z*): [M + H]^+^: calculated for C_18_H_14_N_2_O_2_FS: 341.0760, found: 341.0748.

##### 4.1.2.5 Benzoic Acid [4-(4-Bromo-phenylsulfanyl)-benzylidene]-hydrazide (**2e**)

White powder, mp 186–187 °C, Yield: 94%. IR (cm^–1^) ν 3178 (NH), 3051 (CH, aryl), 1647 (C=O), 1562 (C=N), 1489 (NH), 1350 (C–N), 1242 (Ar–S), 759, 698 (CH, aryl). ^1^H NMR (500 MHz, DMSO) δ: 11.83 (s, 1H), 8.42 (s, 1H), 7.92 (d, *J* = 7.5 Hz, 2H), 7.65 (d, *J* = 8.1 Hz, 2H), 7.61–7.58 (m, 1H), 7.53 (t, *J* = 7.5 Hz, 2H), 7.49 (d, *J* = 8.4 Hz, 2H), 7.15 (d, *J* = 8.1 Hz, 2H), 7.06 (d, *J* = 8.4 Hz, 2H), 3.81 (s, 3H). ^13^C NMR (126 MHz, DMSO) δ: 163.54, 160.57, 147.66, 141.38, 136.50, 133.91, 132.25, 128.94, 128.26, 128.07, 127.47, 122.11, 116.03, 55.81. HRMS (ESI) (*m*/*z*): [M + H]^+^: calculated for C_20_H_16_BrN_2_OS: 411.0167, found: 411.0152.

##### 4.1.2.6 Furan-2-carboxylic Acid [4-(4-Bromo-phenylsulfanyl)-benzylidene]-hydrazide (**2f**)

White powder, mp 190.5–192 °C, Yield: 91%. IR (cm^–1^) ν 3197 (NH), 3047 (CH, aryl), 1651 (C=O), 1589 (C=N), 1489 (NH), 1396 (C–N), 1284 (Ar–S), 825, 748 (CH, aryl). ^1^H NMR (500 MHz, DMSO) δ: 11.82 (s, 1H), 8.39 (s, 1H), 7.94 (s, 1H), 7.62 (d, *J* = 8.3 Hz, 2H), 7.48 (d, *J* = 8.5 Hz, 2H), 7.29 (br s, 1H), 7.14 (d, *J* = 8.2 Hz, 2H), 7.06 (d, *J* = 8.6 Hz, 2H), 6.70 (s, 1H), 3.81 (s, 3H). ^13^C NMR (126 MHz, DMSO) δ: 160.57, 154.60, 147.71, 147.09, 146.30, 141.37, 136.49, 132.16, 128.25, 127.48, 122.09, 116.04, 115.39, 112.55, 55.82. HRMS (ESI) (*m*/*z*): [M + H]^+^: calculated for C_18_H_14_BrN_2_O_2_S: 400.9959, found: 400.9946.

##### 4.1.2.7 Benzoic Acid [4-(4-Methoxy-phenylsulfanyl)-benzylidene]-hydrazide (**2g**)

White powder, mp 181–182 °C, Yield: 91%. IR (cm^–1^) ν 3294 (NH), 3051 (CH, aryl), 1666 (C=O), 1539 (C=N), 1469 (NH), 1365 (C–N), 1273 (Ar–S), 813, 686 (CH, aryl). ^1^H NMR (500 MHz, DMSO) δ: 11.94 (s, 1H), 8.49 (s, 1H), 7.97 (d, *J* = 7.5 Hz, 2H), 7.78 (d, *J* = 8.0 Hz, 2H), 7.66–7.63 (m, 3H), 7.58 (t, *J* = 7.4 Hz, 2H), 7.42 (d, *J* = 8.0 Hz, 2H), 7.39 (d, *J* = 8.2 Hz, 2H). 7.92 (d, *J* = 7.5 Hz, 2H), 7.70 (d, *J* = 8.1 Hz, 2H), 7.61–7.58 (m, 1H), 7.55–7.53 (m, 4H), 7.31 (t, *J* = 8.7 Hz, 2H), 7.27 (d, *J* = 8.1 Hz, 2H). ^13^C NMR (126 MHz, DMSO) δ: 163.59, 147.33, 137.28, 133.95, 133.89, 133.83, 133.75, 133.08, 132.27, 130.83, 128.96, 128.59, 128.10, 121.68. HRMS (ESI) (*m*/*z*): [M + H]^+^: calculated for C_21_H_19_N_2_O_2_S: 363.1167, found: 363.1153.

##### 4.1.2.8 Furan-2-carboxylic Acid [4-(4-Methoxy-phenylsulfanyl)-benzylidene]-hydrazide (**2h**)

White powder, mp 118–119 °C, Yield: 90%. IR (cm^–1^) ν 3228­(NH), 3066 (CH, aryl), 1651 (C=O), 1546 (C=N), 1469 (NH), 1354 (C–N), 1296 (Ar–S), 806, 756 (CH, aryl). ^1^H NMR (500 MHz, DMSO) δ: 11.89 (s, 1H), 8.44 (s, 1H), 7.95 (s, 1H), 7.71 (d, *J* = 8.2 Hz, 2H), 7.60 (d, *J* = 8.4 Hz, 2H), 7.36 (d, *J* = 8.2 Hz, 2H), 7.33 (d, *J* = 8.4 Hz, 2H), 6.71 (s, 1H). ^13^C NMR (126 MHz, DMSO) δ: 154.65, 147.39, 147.06, 146.36, 137.27, 133.96, 133.80, 133.70, 133.07, 130.83, 128.57, 121.67, 115.51, 112.57. HRMS (ESI) (*m*/*z*): [M + H]^+^: calculated for C_19_H_17_N_2_O_3_S: 353.0960, found: 353.0947.

### 4.2 Biology

#### 4.2.1 Chemicals and Cell Culture

All compounds were dissolved in dimethyl sulfoxide (DMSO) at a stock concentration of 50 mM. Cisplatin was used as a reference drug (a clinically used one) at the dose of the plasma peak level. Further dilutions were made in the cell culture medium. Human lung cancer (A549), breast cancer (MDA-MB-231), hepatocellular carcinoma (HepG2), colon cancer (HCT116), and normal bronchial epithelium (BEAS-2B) cell lines were cultured with 1% v/v penicillin/streptomycin and 10% fetal bovine serum-enriched Dulbecco’s modified Eagle’s medium (DMEM). All of the cells were incubated at 37 °C in a 5% v/v CO2 environment.

#### 4.2.2 Cell Viability Assay

A sulforhodamine B (SRB) assay was used for viability. The cells were seeded in 96-well plates at 5 × 10^3^ cell density and treated with 10 μM for each compound for 48 h. Cells were in situ fixed with trichloroacetic acid at 4 °C for 1 h following treatment. Then, wells were washed 5 times with deionized water. A 0.4% SRB solution was added into each well and incubated at room temperature for 30 min, followed by washing with 1% acetic acid to eliminate nonspecific bindings and unbounded dye. The bounded SRB dye was dissolved by adding 10 mM Tris base. Absorbances were read at 564 nm using a Lumistar Omega microplate reader. For further experiments, the growth rate assessment was performed with the SRB assay, in which more detailed information (e.g., antiproliferative, cytostatic, or cytotoxic doses/effects of the compound) is obtained.^90^

#### 4.2.3 In Ovo Experiments

##### 4.2.3.1 Assessment of the Antitumoral Effect

Fertilized chicken eggs of the ROSS308 strain were incubated at 37 °C with 60% humidity until day 3 (EDD3). On EDD3, 2 mL of albumen was aspirated from each egg, and the eggs were returned to the incubator. On EDD4, a circular window approximately 1–2 cm in diameter was opened on the upper surface of each egg, covered with adhesive tape, and incubation was continued. Embryo viability was monitored daily throughout the incubation period. Tumor cells were prepared at a concentration of 1 × 10^6^ cells and mixed with Matrigel in a centrifuge tube. The Matrigel–cell mixture was then grafted onto the chorioallantoic membrane (CAM) of eggs at EDD8. On EDD12, compound **2c** was applied topically onto the tumor surface at its IC_50_ concentration in a volume of 50 μL. In EDD14, tumors were photographed, and tumor volumes were analyzed using three different methods. Tumor growth was quantitatively evaluated using three complementary approaches. All photographs were captured under identical magnification settings, and all images were normalized to the same scale prior to analysis.^91,92^

##### 4.2.3.2 Image-Based Area Measurement

Tumor areas were measured from captured photographs using ImageJ software. The pixel-based surface areas were converted to mm^2^ according to the image scale.

##### 4.2.3.3 Ellipsoid Volume Estimation

Tumor length (*L*) and width (*W*) were measured from the photographs, and tumor volume was calculated using the following formula:^93–95^

$$V=\frac{W\times L^{2}}{2}$$

##### 4.2.3.4 Spherical Volume Estimation

Tumor dimensions were determined from digital photographs by measuring the long (*L*) and short (*W*) axes of each tumor. The radius (*r*) of the assumed spherical tumor was calculated as half of the geometric mean of the two diameters:

$$r=\frac{\sqrt{W\times L}}{2}$$

The tumor volume (*V*) was then calculated according to the formula for a sphere:

$$V=\frac{4}{3}\pi r^{2}$$

This method provides a 3D approximation of tumor size based on surface measurements obtained from standardized, scale-normalized images.

##### 4.2.3.5 Assessment of the Antiangiogenic Effect

ROSS308 fertilized chicken eggs were incubated in 60% humidity at 37 °C until embryonic development day 3 (EDD3). At EDD3, 2 mL of albumen was withdrawn from the egg. On EDD4, the upper part of the eggs was opened in a round shape with a diameter of 1–2 cm and sealed with a plaster. The viability of the embryos was monitored over the following days. On EDD6, **2c** (1000μM, 100μM, 10μM) was applied topically in 50 μl (with a minimum of 5 eggs per group) onto the CAM layer. Photographs of the CAM layer were taken immediately after application (0 h) and again 24 and 48 h later.^91,92^ These images were then analyzed according to the scoring table below.^96^

#### 4.2.4 Target Prediction and Docking Studies

By using the 2D chemical structure of compound **2c**, the target prediction was performed by using the Way2Drug platform. Followed by the initial screening of potential targets for compound **2c**, the CLC-Pred (Cell Line Cytotoxicity Predictor) 2.0 web service tool^35^ was used to establish the relationship between compound **2c** and its possible molecular targets by setting the cutoff value for *p*-values as 0.4. The target pool was subjected to the GEPIA2 tool to check the expression levels of nontumor and tumor LIHC tissues in liver hepatocellular carcinoma (LIHC).^36^ The molecular targets having different expression patterns in nontumor and LIHC tissues (*p*-val< 0.05) were subjected to docking simulations. To retrieve the possible protein–protein interactions within predicted targets, the lists of targets are given as an input for the STRING-db tool to create protein–protein interaction networks by using the default parameters.^97^ For all receptors, 3D protein models were retrieved from the Protein Data Bank. To obtain the 3D structure of compound **2c**, its SMILES formula (FC1=CC=C­(SC2=CC=C­(\C=N\NC­(–O)­C3=CC=CC=C3)­C=C2)­C=C1) was first used to generate its 3D structure by employing Phyton3, RDKit.^98^ The preparation of ligand and receptor structures was also performed by AutoDock to convert .pdb files to .pdbqt formats, including partial charge and atom type. A docking study was conducted with the AutoDock4 program with AutoDock version 1.5.7.^99^ The receptor was kept as a rigid, but the ligand was treated as flexible during docking. The grid box, including the active site of each receptor, was employed to run docking. Default options were employed, but in particular, the grid spacing was set to 0.375 Å. All results were analyzed according to the poses and scores obtained by Vina. The contact analysis of docking outputs was run by Chimera after the selection of the ligand–receptor complex in PyMOL. The final visualization of ligand–receptor conformations was drawn in Visual Molecular Dynamics. The predicted inhibition values for compound **2c** to those targets were theoretically calculated for each selected target’s pose by using the Cheng–Prusoff equation, which assumes competitive inhibition.^100,101^

#### 4.2.5 Statistical Analysis

Tumor size measurements obtained from the in ovo model were analyzed using one-way analysis of variance (one-way ANOVA) to evaluate overall differences among the control and treatment groups. The assumptions of normality and homogeneity of variances were assessed using the Shapiro–Wilk and Levene tests, respectively. When a significant main effect was detected by ANOVA, Dunnett’s posthoc test was performed to identify which treatment groups differed significantly from the control group. Antiangiogenic effects were evaluated by analyzing treatment- and time-dependent changes in vascular development using a two-way analysis of variance (two-way ANOVA). This test was used to assess the independent effects of treatment and time (or dose, depending on your experiment), as well as the potential interaction between these two factors on angiogenesis parameters. All results are expressed as the mean ± SEM, and statistical significance was accepted at *p* < 0.05.

## Supplementary Material



## Notes

Experimental details, along with copies of the ^1^H NMR, ^13^C NMR, and HRMS spectra for all compounds, as well as the pose and pose-specific docking score details of compound **2c** (PDF)
