# Supplementary material for: Evaluation of Anticancer Activity of Novel Sulfanyl-Substituted Hydrazone Compounds in Hepatocellular Carcinoma: In Vitro, In Silico, and In Ovo Studies
Source: ACS Omega. 2026 Mar 5;11(10):16657–72. doi: 10.1021/acsomega.5c13125 (PMC13000631; doi:10.1021/acsomega.5c13125)
Supplement: Supplementary file 1 [file ao5c13125_si_001.pdf]

Supplementary data

Evaluation of Anticancer Activity of Novel Sulfanyl-Substituted Hydrazone Compounds in Hepatocellular Carcinoma: In Vitro, In Silico, and In Ovo Studies

Hatice BAŞPINAR KÜÇÜK[a]*, Tülay YILDIZ[a], Yaren ARASAN[c], Buse Meriç AÇAR[e], Duygu KURTOĞLU[c], Aslı KUTLU[c,d], Remzi Okan AKAR[b,c], Sinem KILIÇ[c], Demet GÜL ERYILMAZ[c], Engin ULUKAYA[b,c]*

[a]Istanbul University-Cerrahpasa, Faculty of Engineering, Department of Chemistry, Organic Chemistry Division, Avcılar, Istanbul, 34320, Türkiye
[b]İstinye University, Faculty of Medical School, Department of Medical Biochemistry, İstanbul, 34396, Türkiye
[c]Molecular Cancer Research Center (ISUMKAM), Istinye University, Istanbul, 34396, Türkiye
[d]Istinye University, Faculty of Engineering and Natural Science, Department of Molecular Biology and Genetics, İstanbul, 34396, Türkiye
[e]Istinye University, Graduate School of Education, Doctoral Program in Molecular Oncology, Istanbul, 34396, Türkiye

*Corresponding authors: Hatice BAŞPINAR KÜÇÜK email: baspinar@iuc.edu.tr; Engin ULUKAYA email: eulukaya@istinye.edu.tr

CONTENTS:

1. Chemistry S2
2. ^{1}H NMR, ^{13}C NMR, and HRMS spectra of all compounds S3-S18
3. Pose and score details of compound **2c** S19-S25
4. References S26

General

All the starting materials were commercially available reagents and used without further purification. Solvents for chromatography were of technical grade and distilled prior to use. Melting points were recorded with Buchi melting point B-540 and uncorrected. ^{1}H and ^{13}C NMR spectra were recorded at 500 MHz for ^{1}H and at 125 MHz for ^{13}C using Me_4Si as the internal standard in DMSO with Bruker Avance NEO 500 MHz NMR spectrometer. Coupling constants were given in hertz (Hz). HRMS were recorded on Thermo Scientific™ Q Exactive Hybrid Quadrupole-Orbitrap MS. IR spectra were recorded on a Agilent Cary 630 FTIR Spectrometer. All reactions were monitored by thin-layer chromatography (TLC) using silica-gel plates (silica gel 60 F254 0.25 mm).

General experimental procedure of 4-phenylsulfanyl-benzaldehydes (1a-d) [1]

To a solution of DMF (10 mL) containing 4-fluorobenzaldehyde (5.0 mmol) and thiophenol (5.0 mmol) was added K_2CO_3 (5.0 mmol) and the reaction mixture was stirred for 2 h at 155 °C under an nitrogen atmosphere. It was cooled to room temperature and after usual workup and concentration, the product was purified over silica gel.

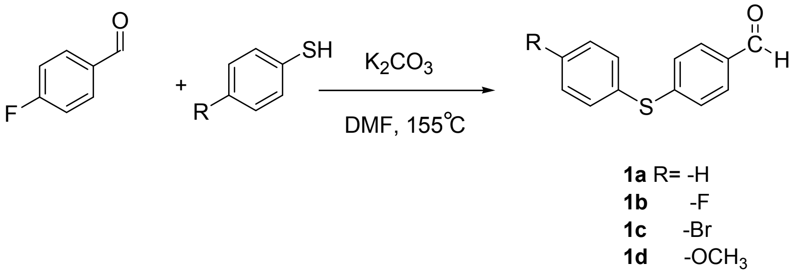

Scheme S1. The Ullmann type reaction of 4-fluorobenzaldehyde and thio phenol derivatives.

General experimental procedure of hydrazones (2a-2h) [2]

To a solution of appropriate hydrazide (benzoic acid hydrazide or furan-2-carboxylic acid hydrazide) (2.0 mmol) in absolute ethanol a stirred solution of substituted 4-phenylsulfanyl-benzaldehydes **1a-d** (2.0 mmol) was added. The solution was refluxed for 2–3h. The solid product formed was collected by filtration and recrystallized with ethanol.

NMR and HRMS Spectra of all the reported compounds:

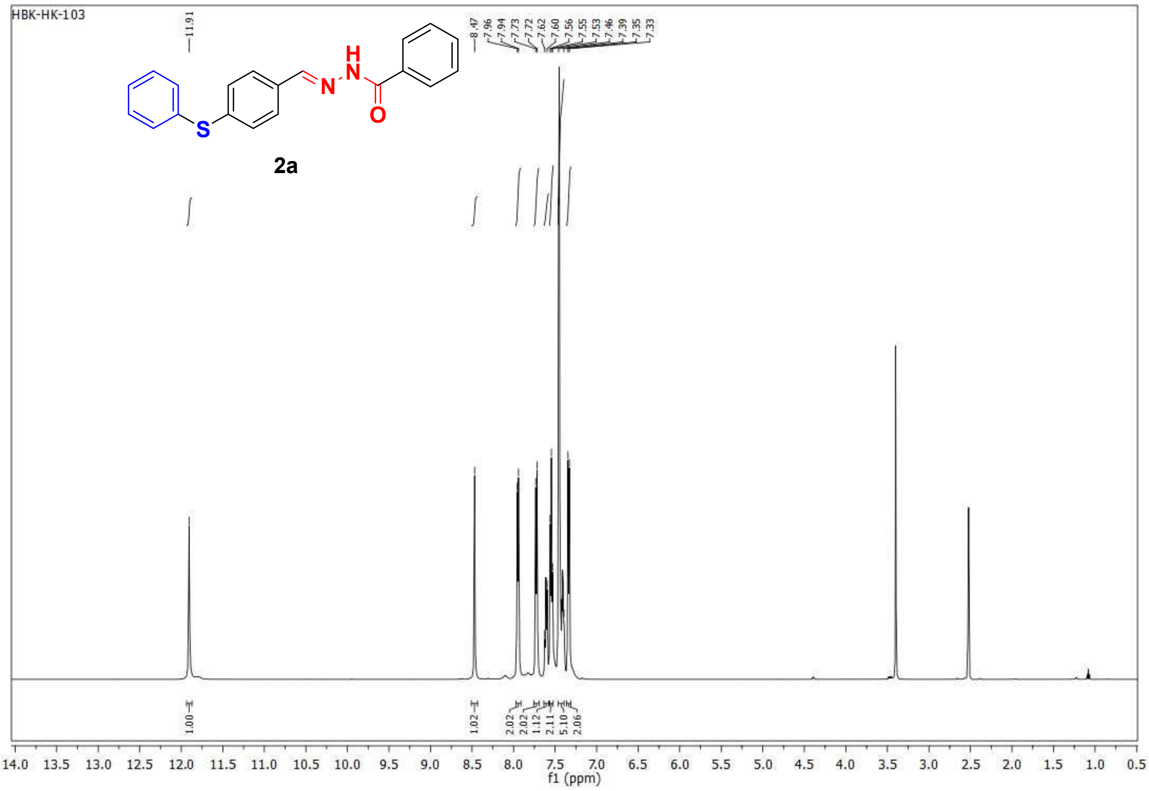

Figure S1. ^{1}H-NMR spectra of compound **2a**.

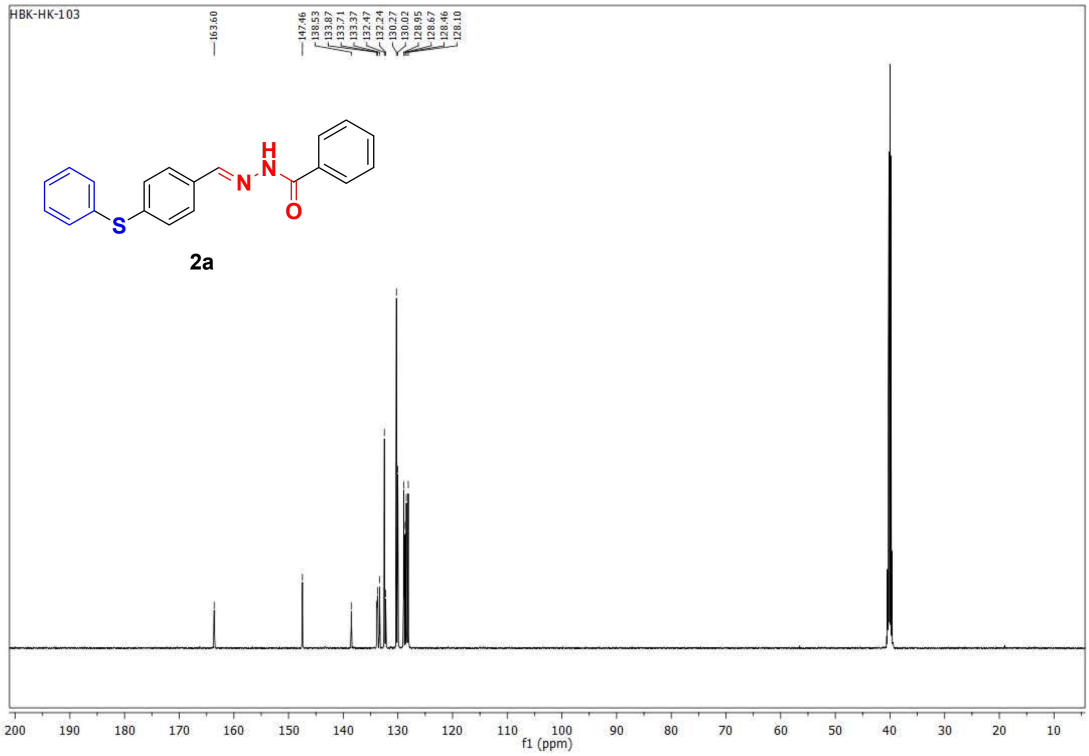

Figure S2. ^{13}C-NMR spectra of compound **2a**.

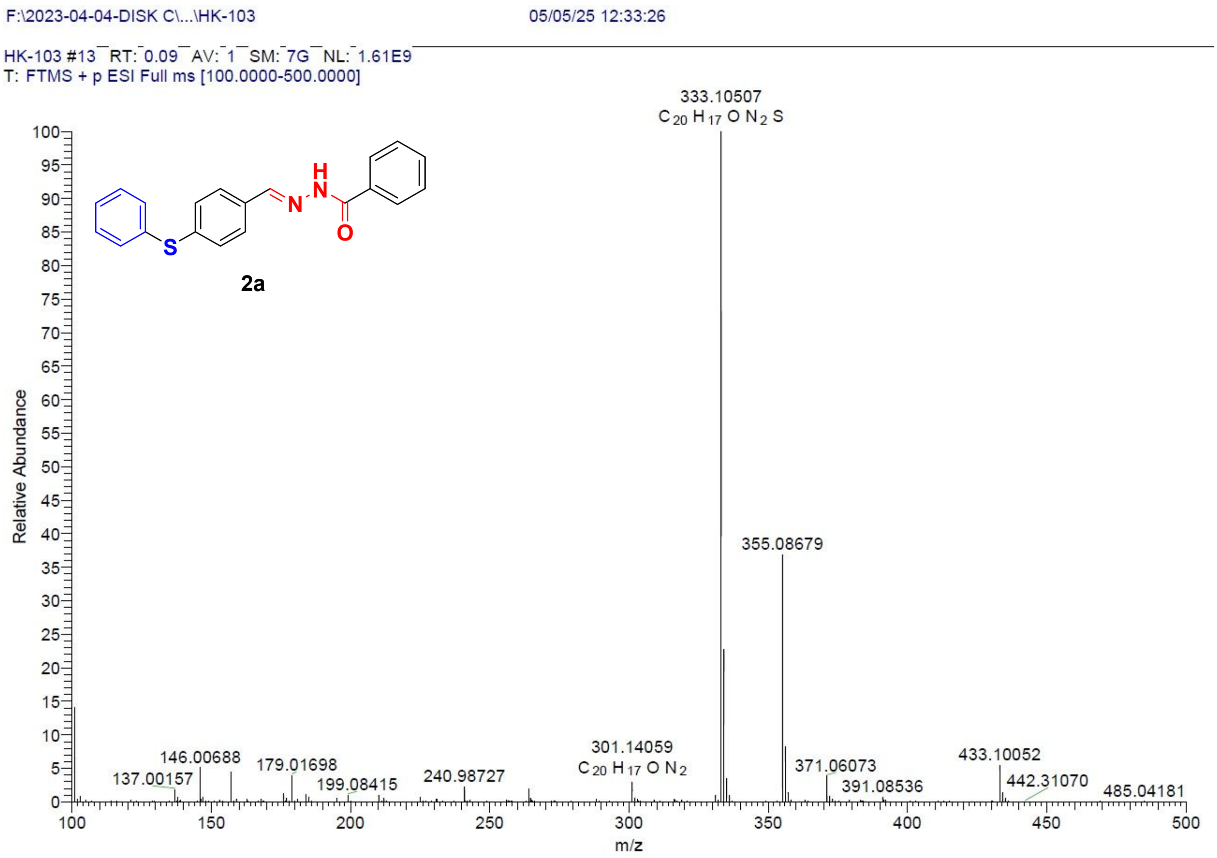

Figure S3. HRMS spectra of compound **2a**.

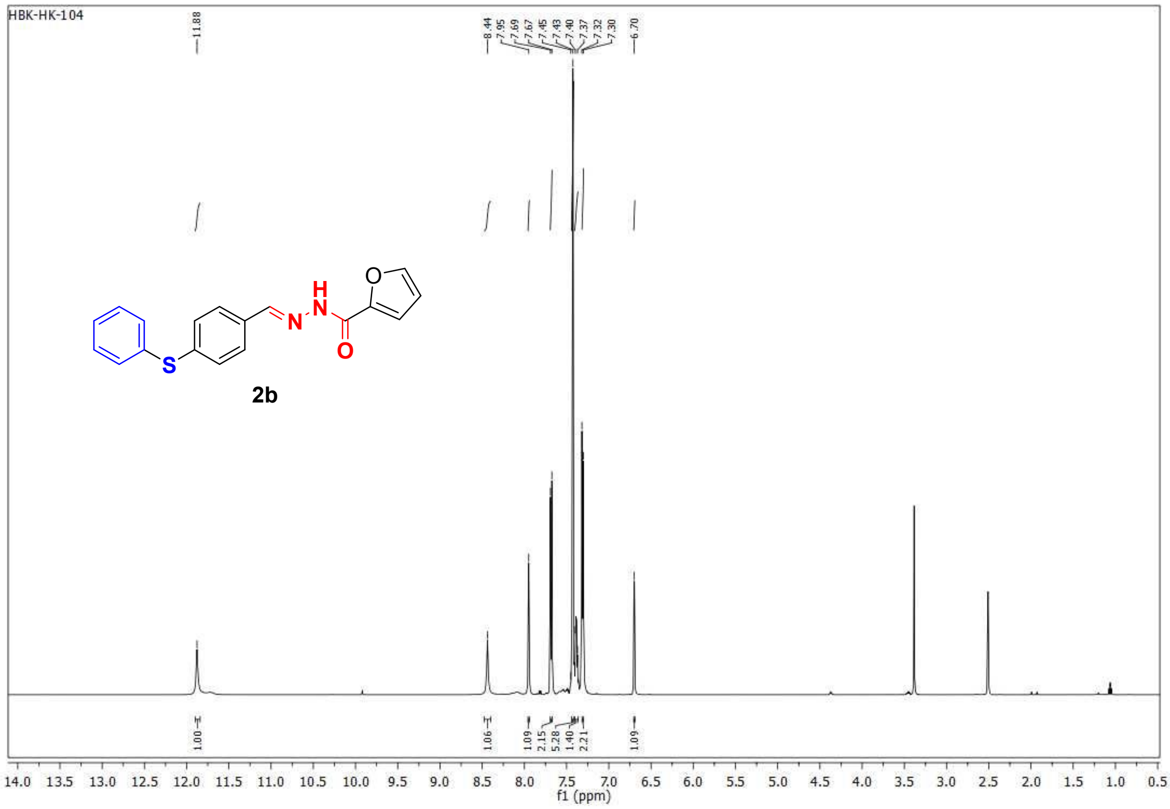

Figure S4. ^{1}H-NMR spectra of compound **2b**.

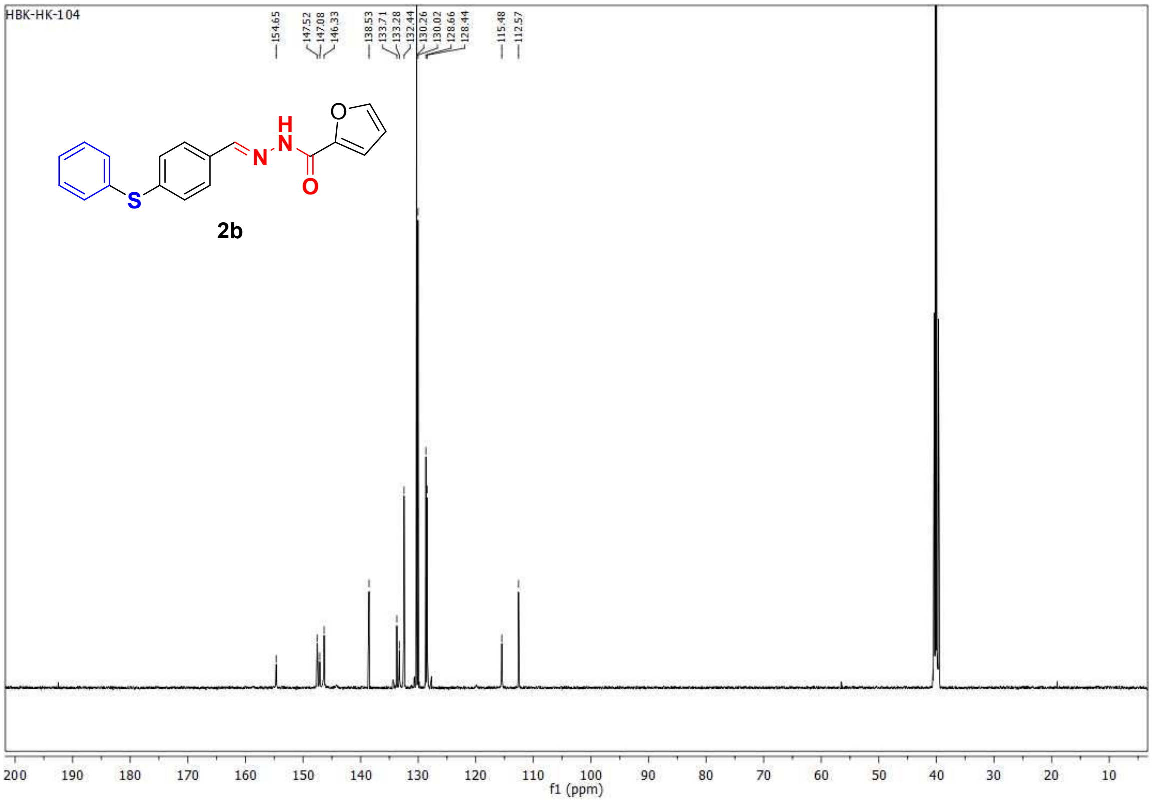

Figure S5. ^{13}C-NMR spectra of compound **2b**.

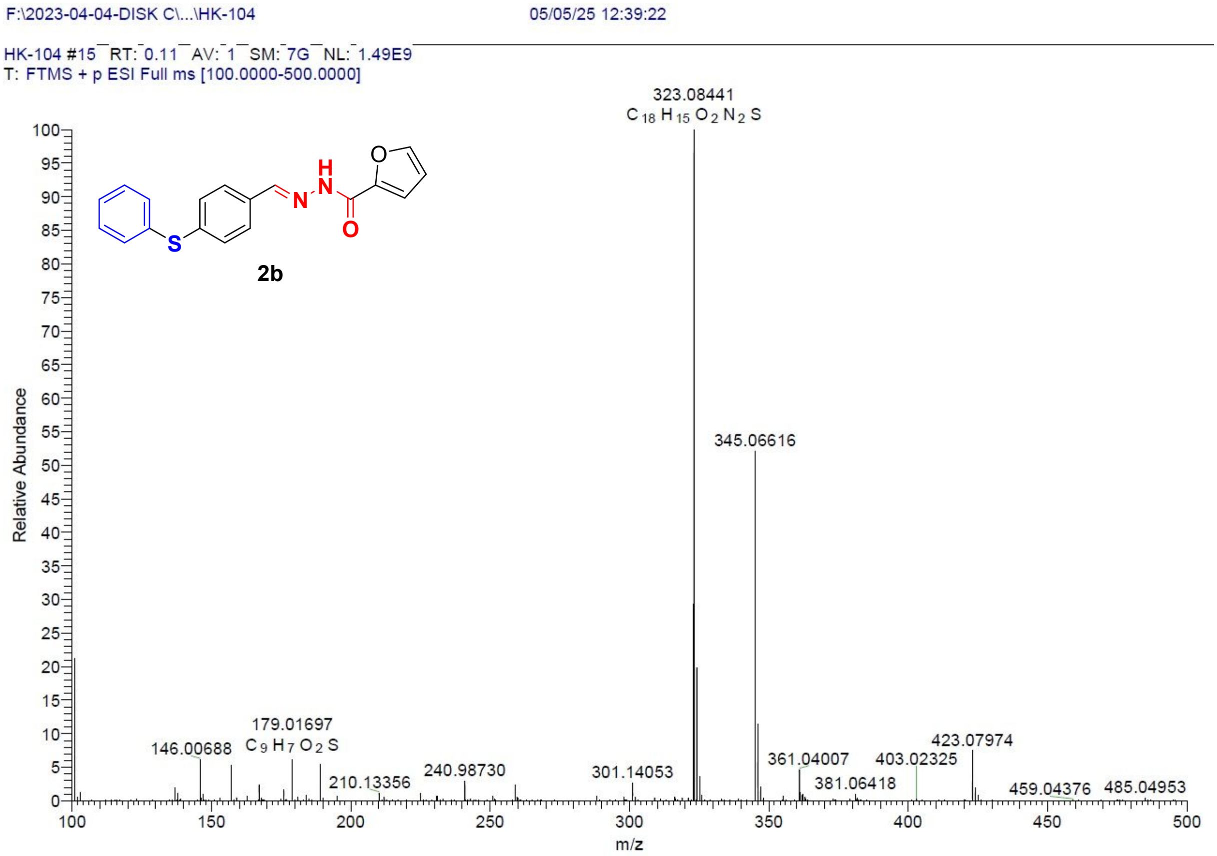

Figure S6. HRMS spectra of compound **2b**.

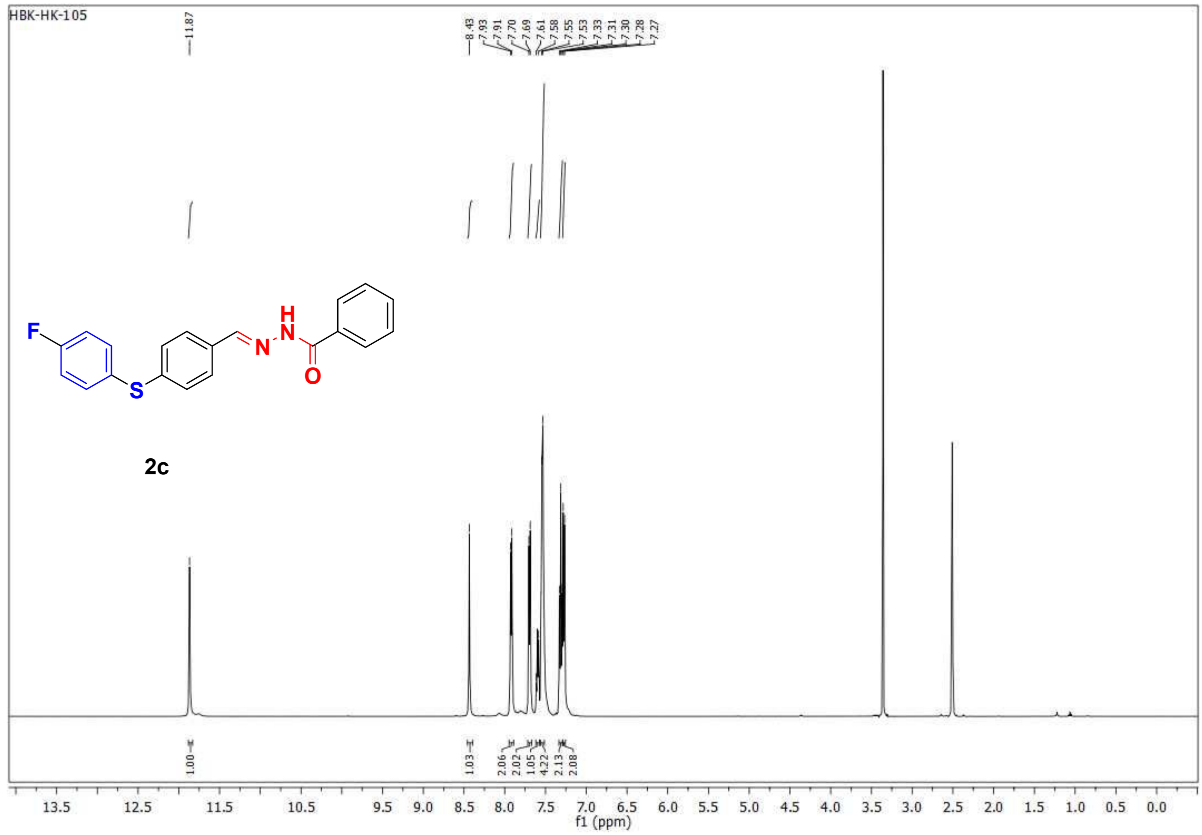

Figure S7. ^{1}H-NMR spectra of compound **2c**.

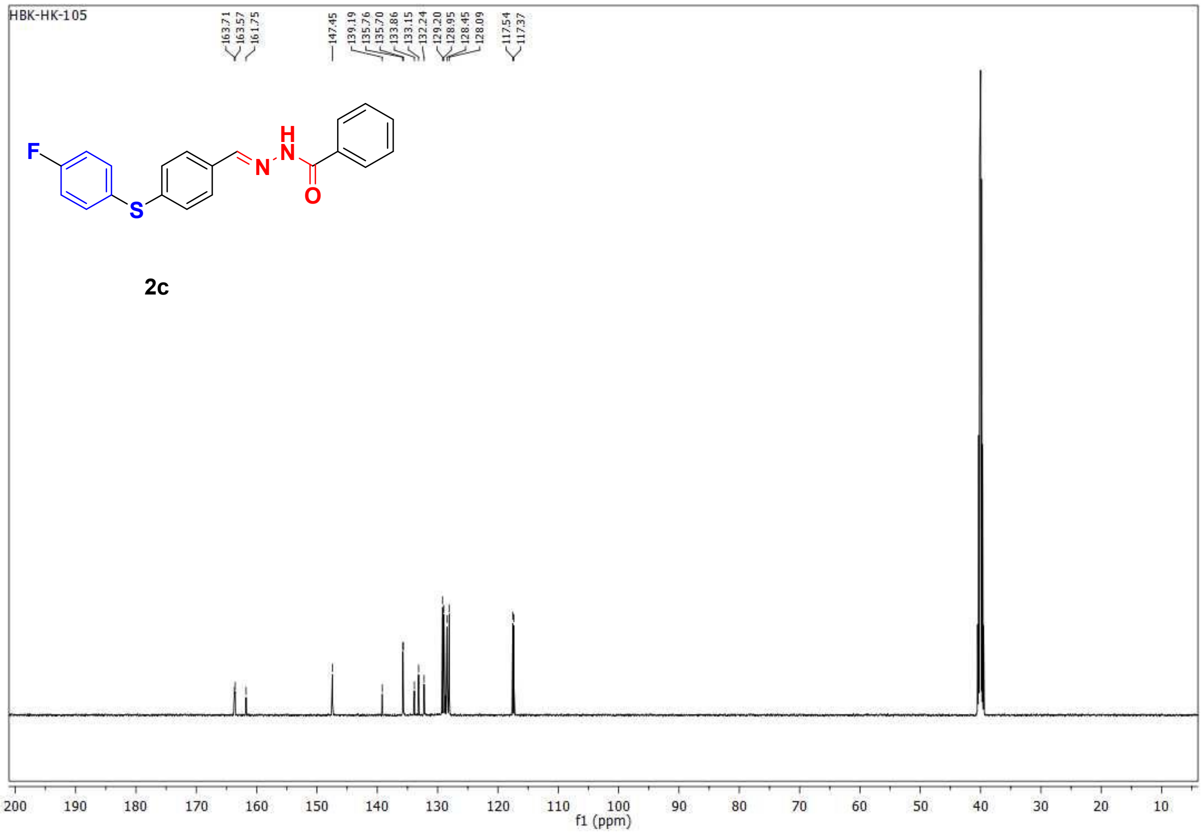

Figure S8. ^{13}C-NMR spectra of compound **2c**.

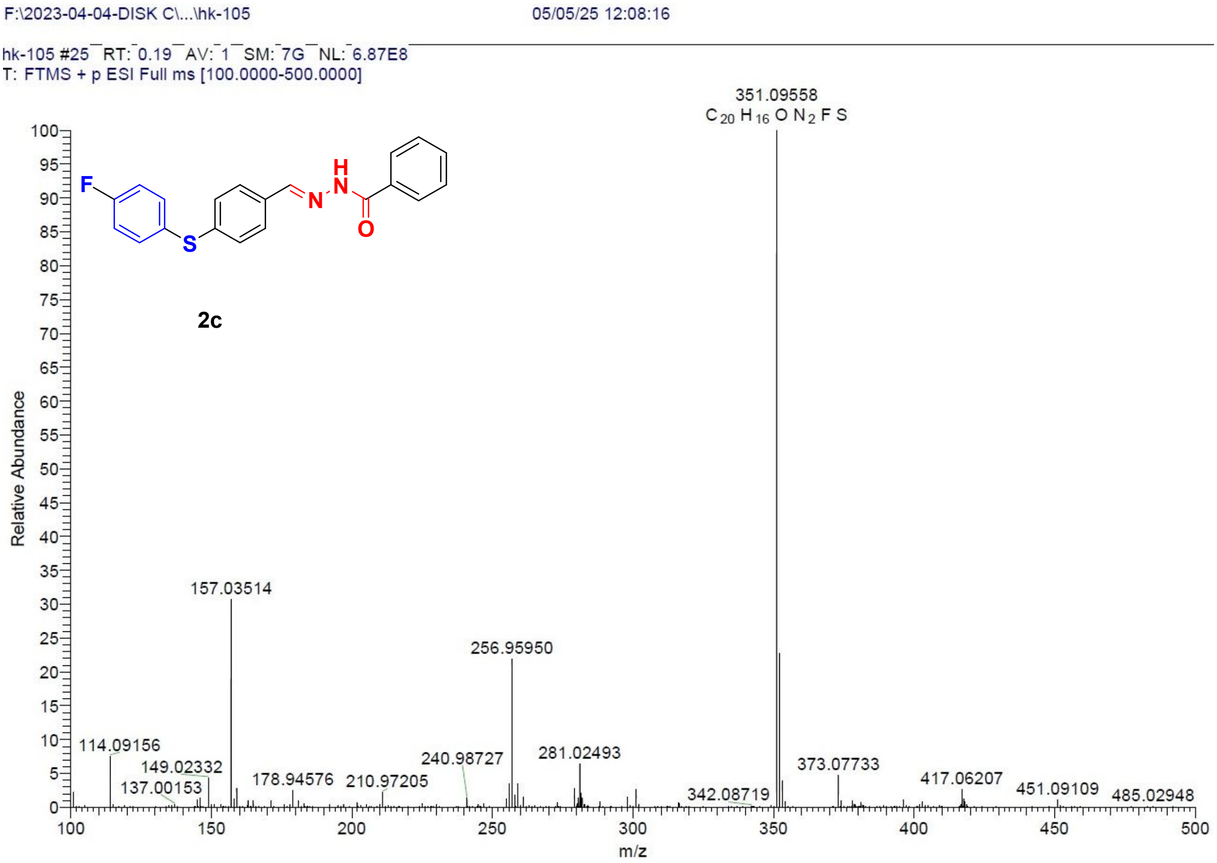

Figure S9. HRMS spectra of compound **2c**.

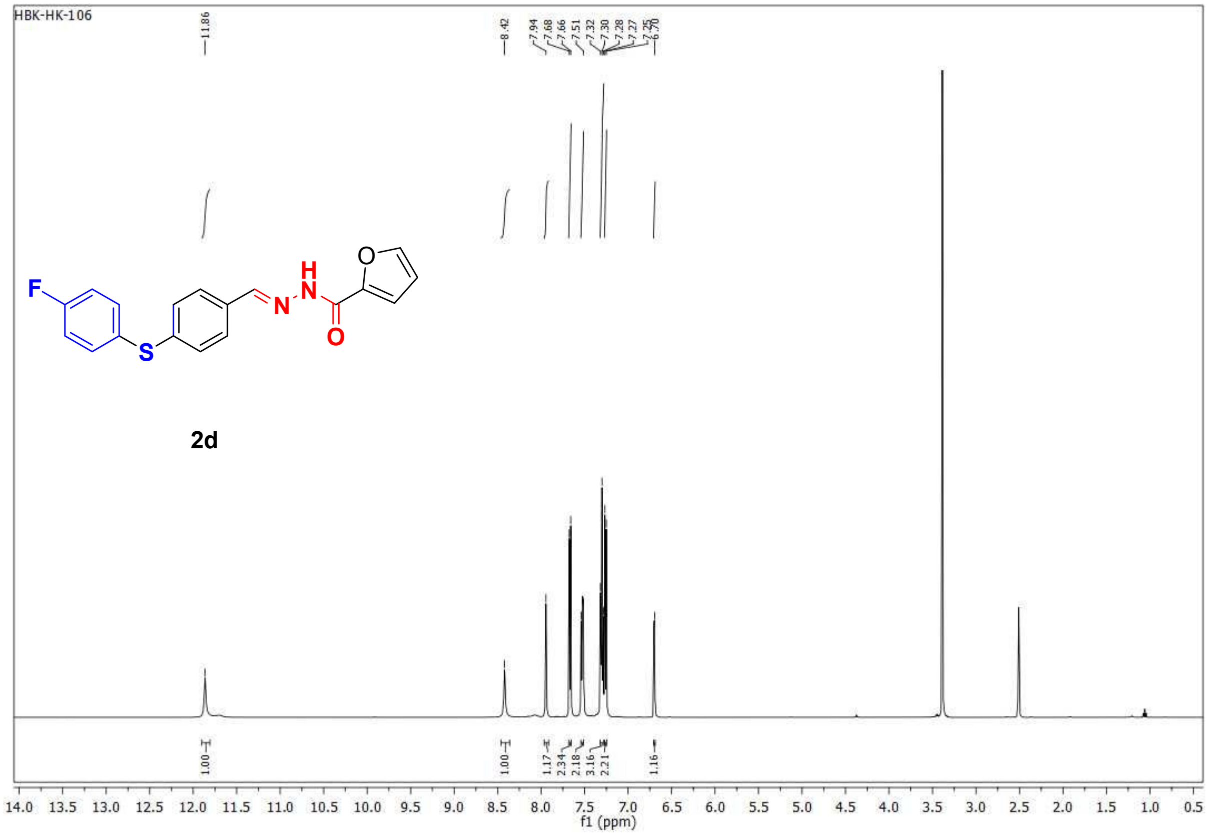

Figure S10. ^{1}H-NMR spectra of compound **2d**.

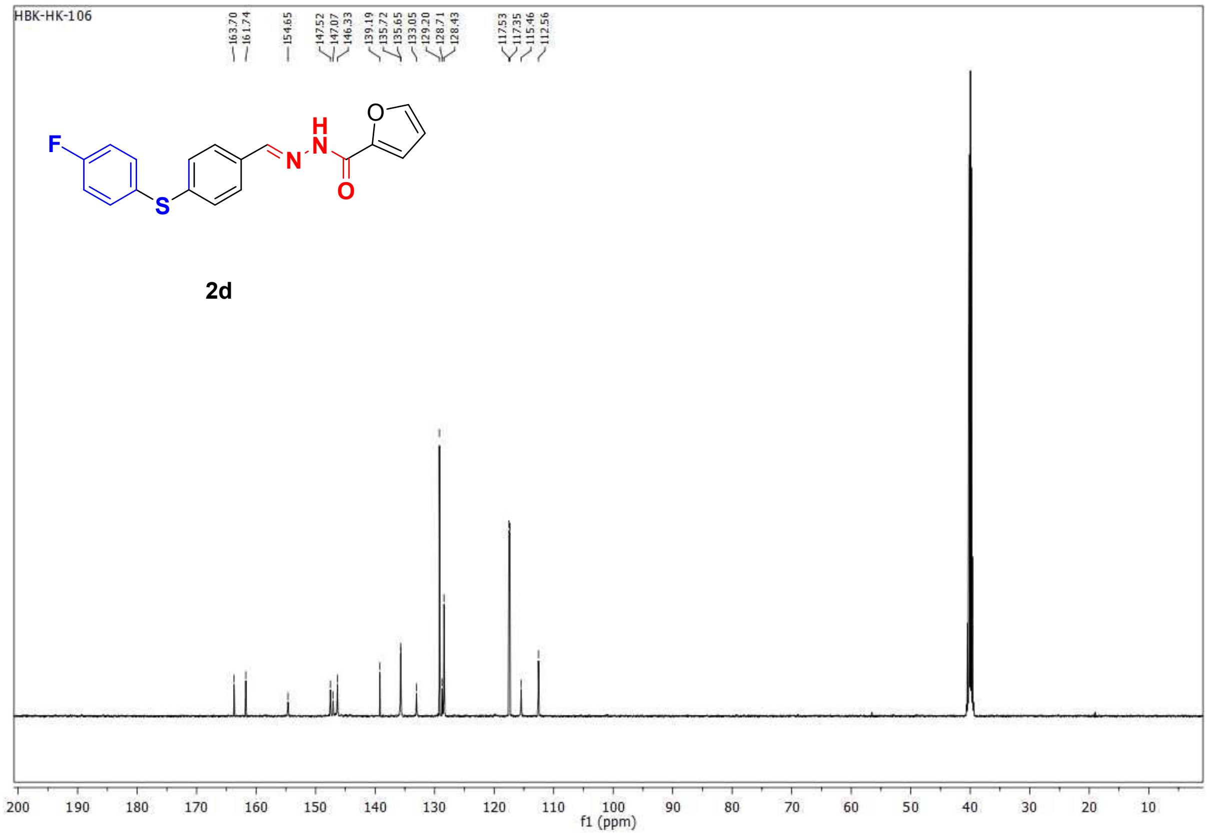

Figure S11. ^{13}C-NMR spectra of compound **2d**.

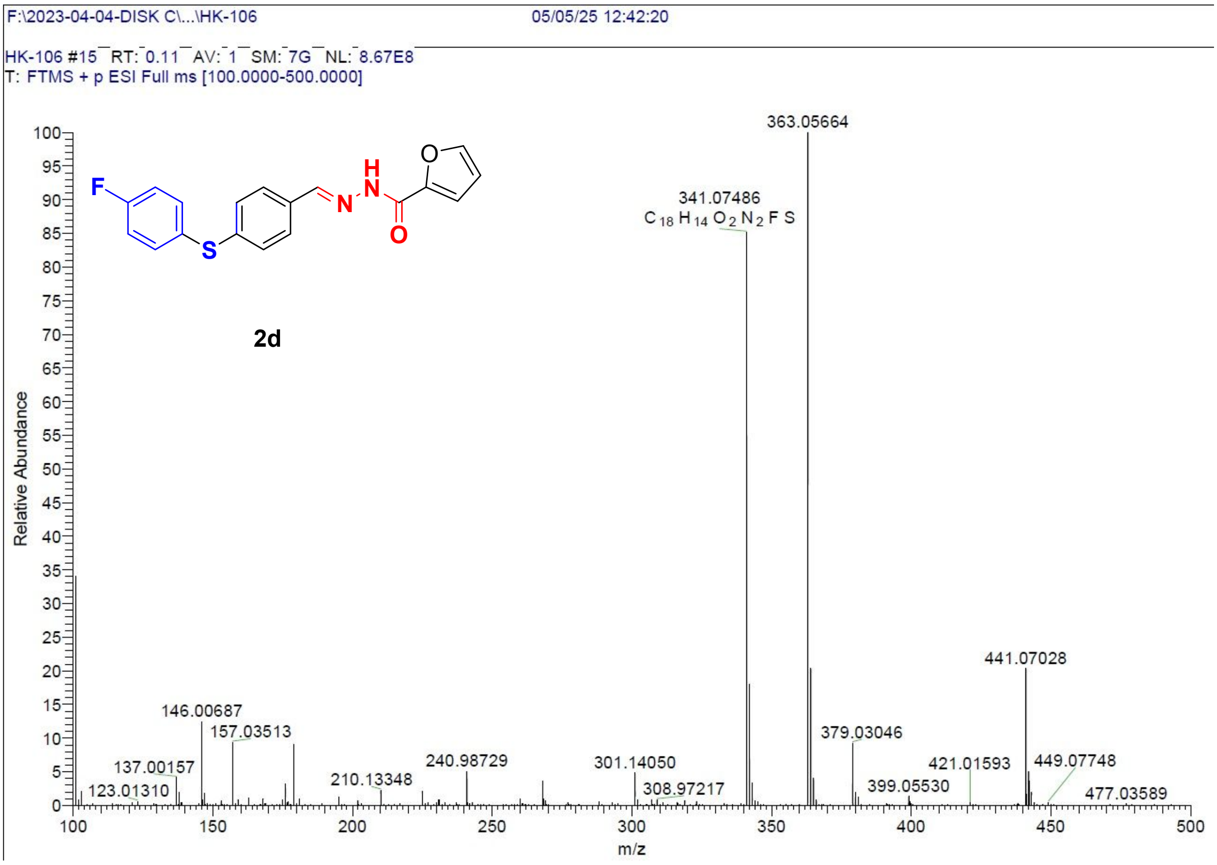

Figure S12. HRMS spectra of compound **2d**.

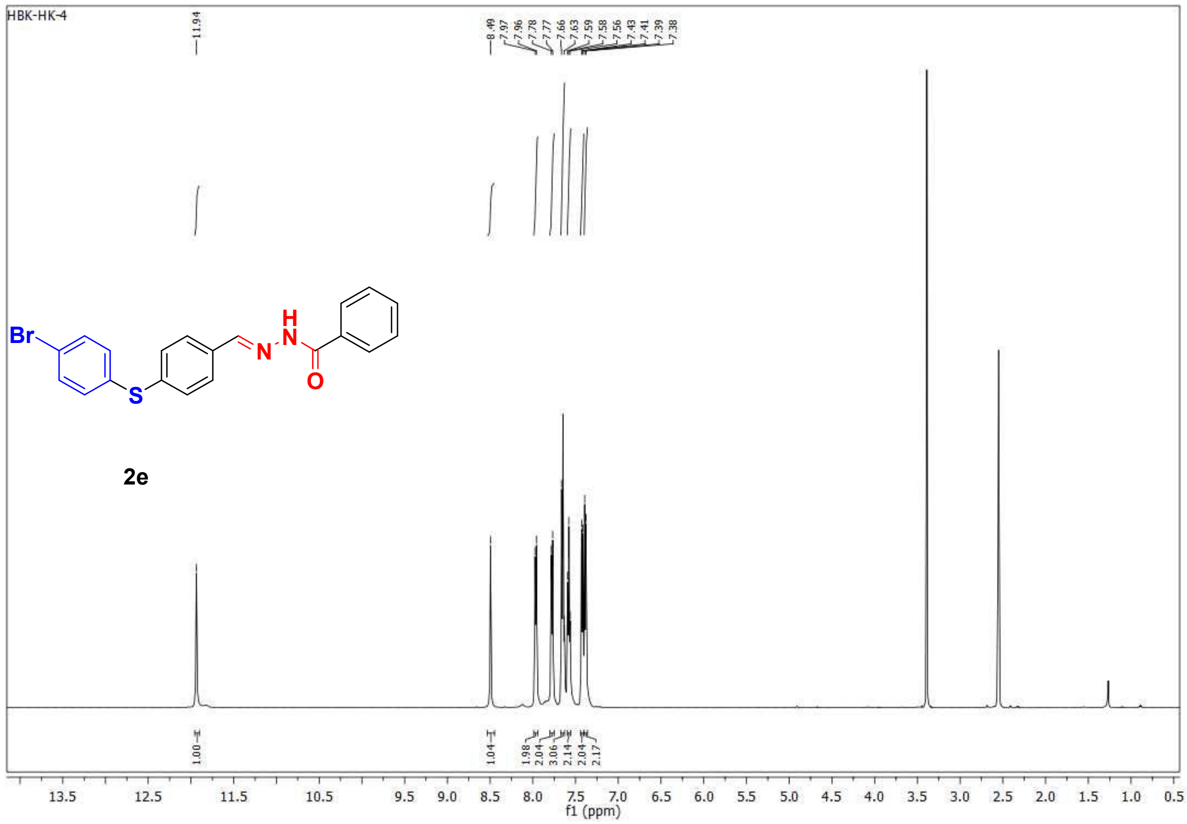

Figure S13. ^{1}H-NMR spectra of compound **2e**.

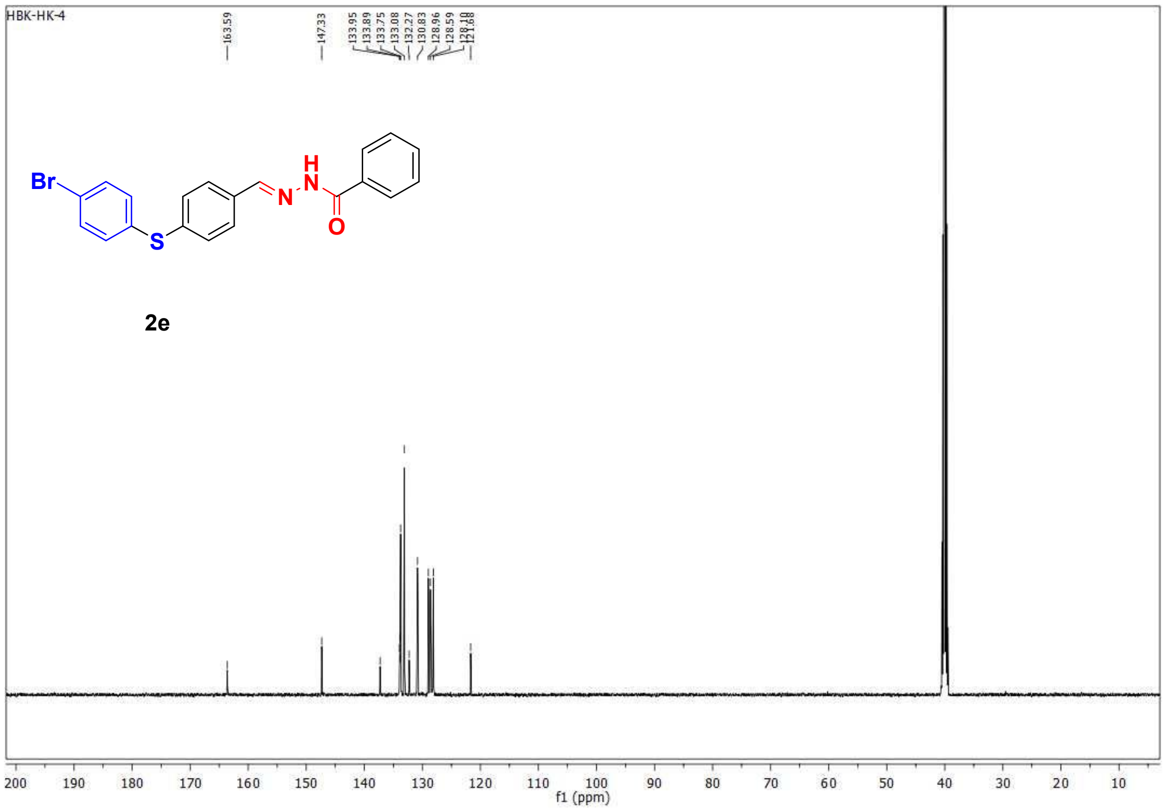

Figure S14. ^{13}C-NMR spectra of compound **2e**.

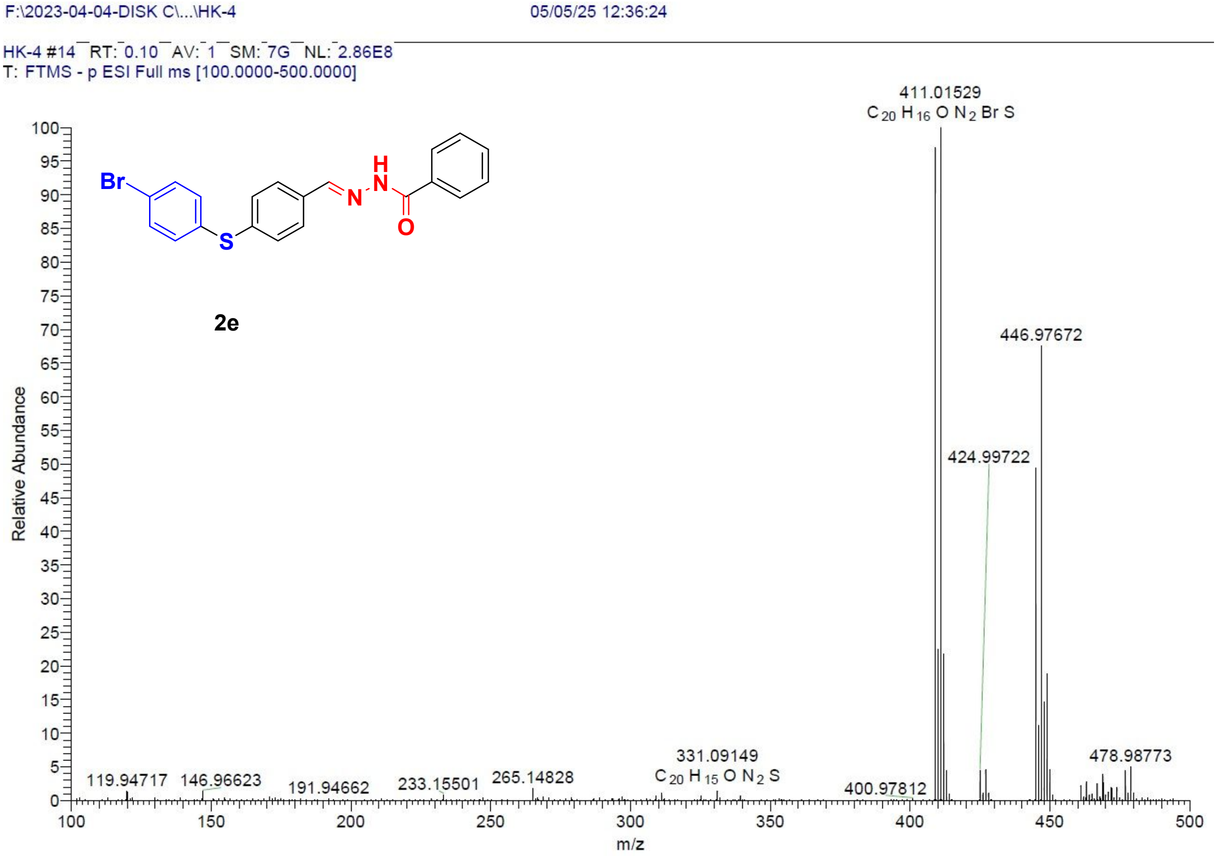

Figure S15. HRMS spectra of compound **2e**.

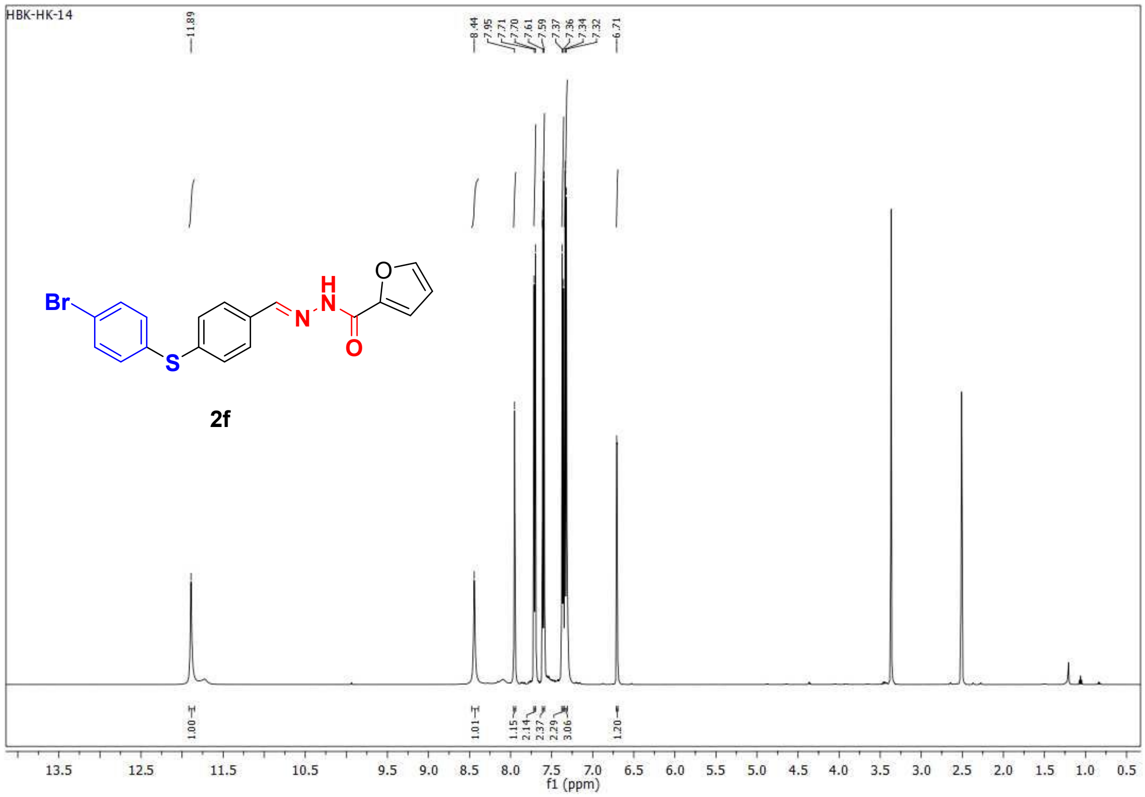

Figure S16. ^{1}H-NMR spectra of compound **2f**.

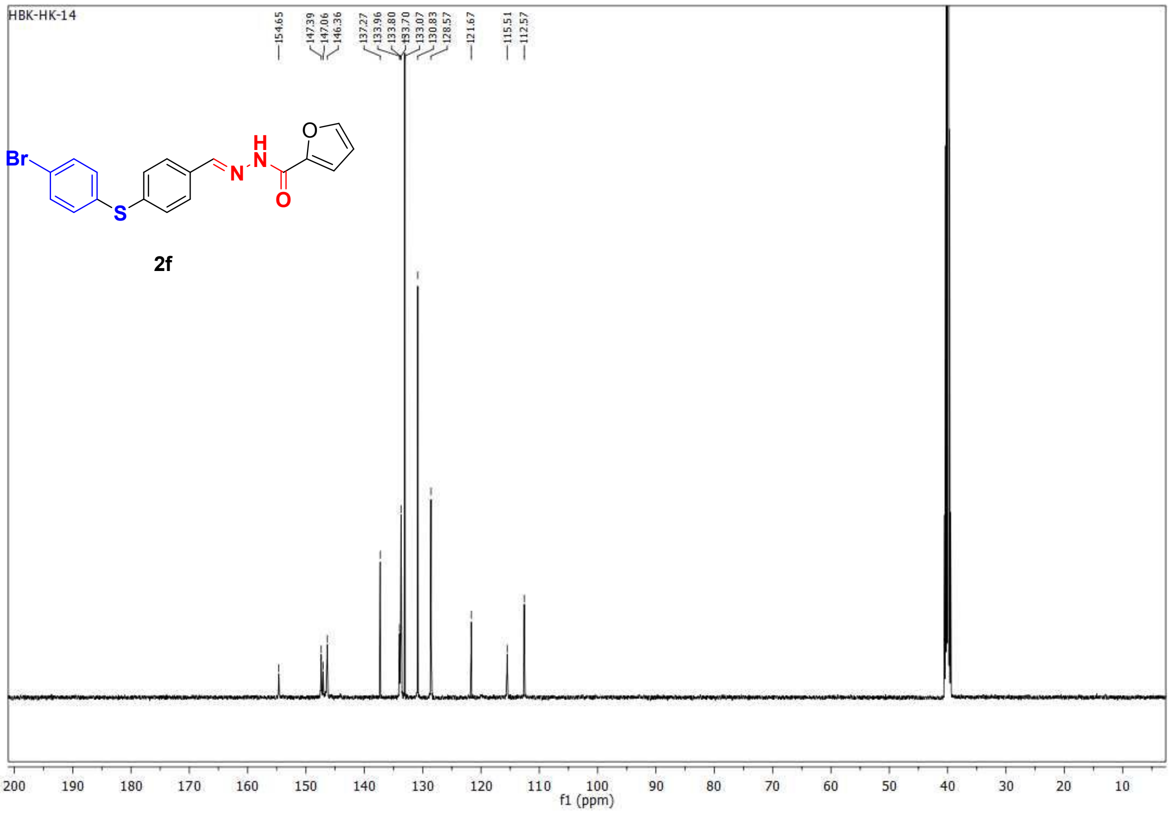

Figure S17. ^{13}C-NMR spectra of compound **2f**.

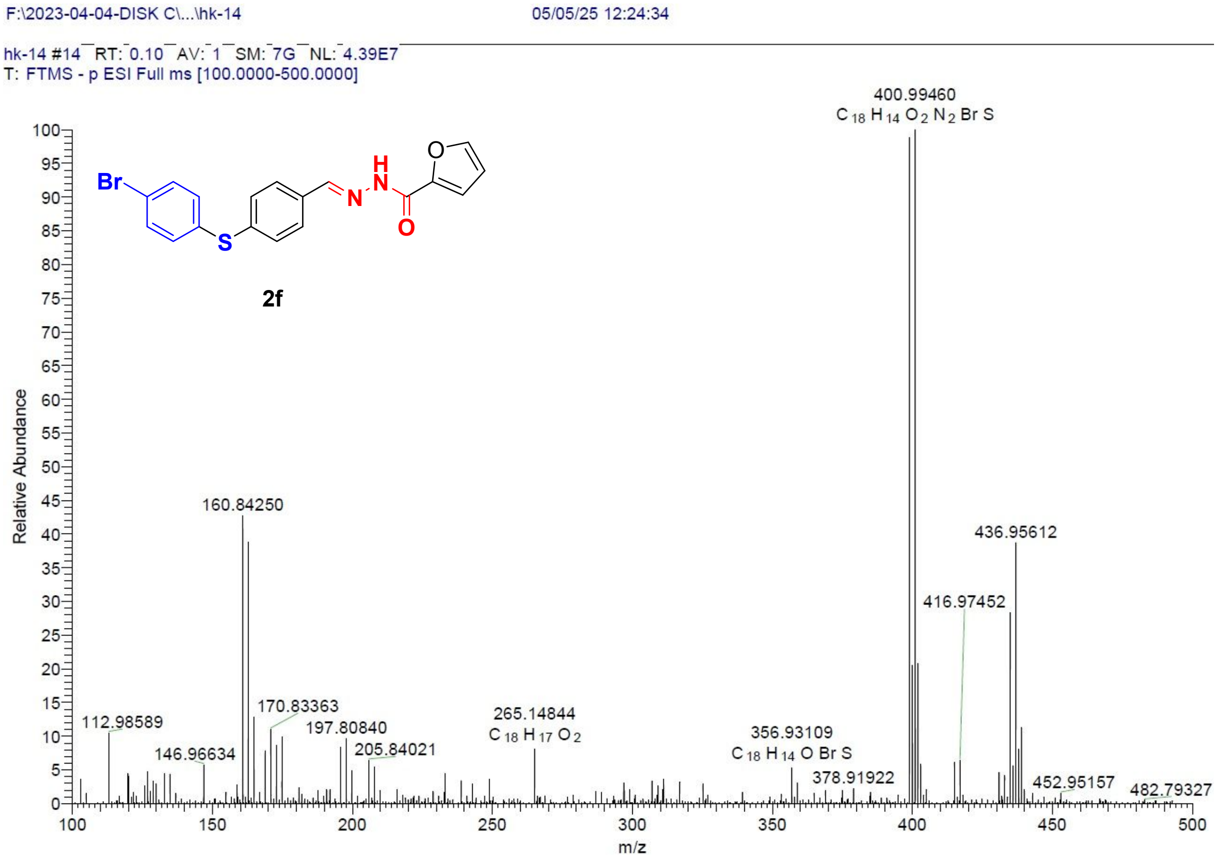

Figure S18. HRMS spectra of compound **2f**.

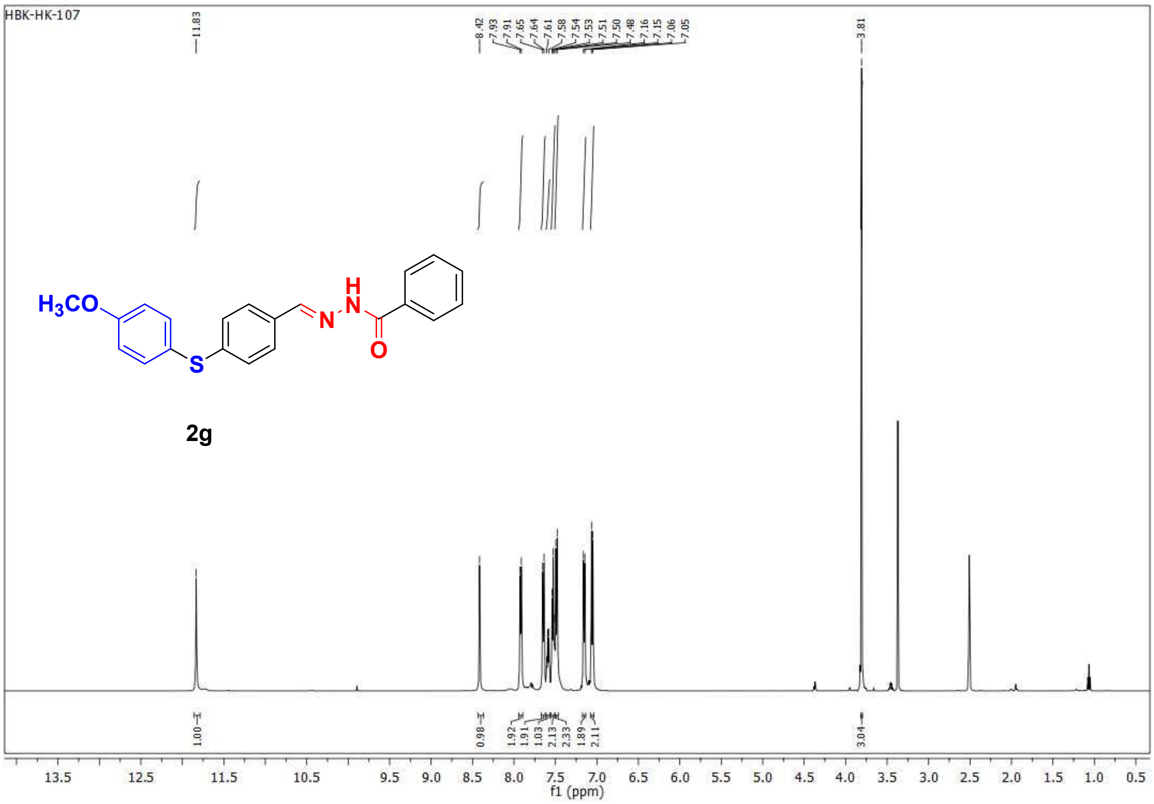

Figure S19. ^{1}H-NMR spectra of compound **2g**.

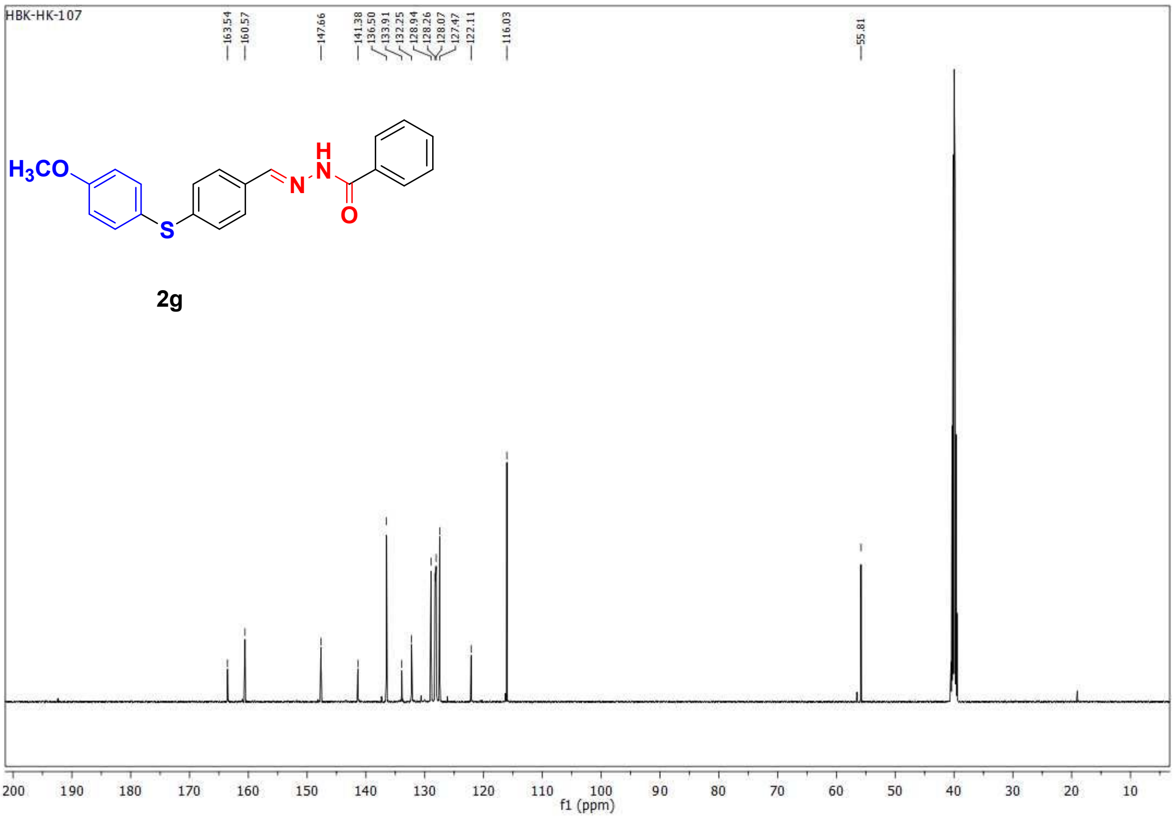

Figure S20. ^{13}C-NMR spectra of compound **2g**.

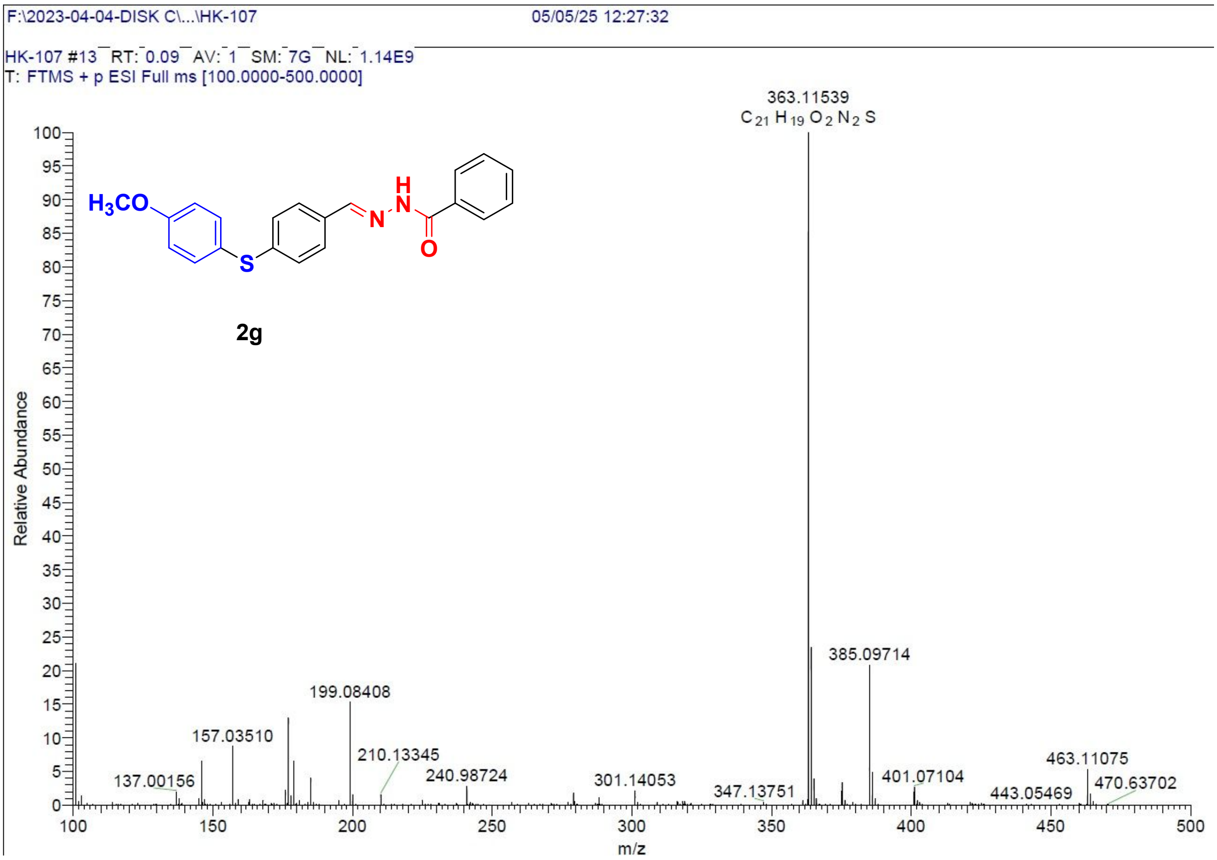

Figure S21. HRMS spectra of compound **2g**.

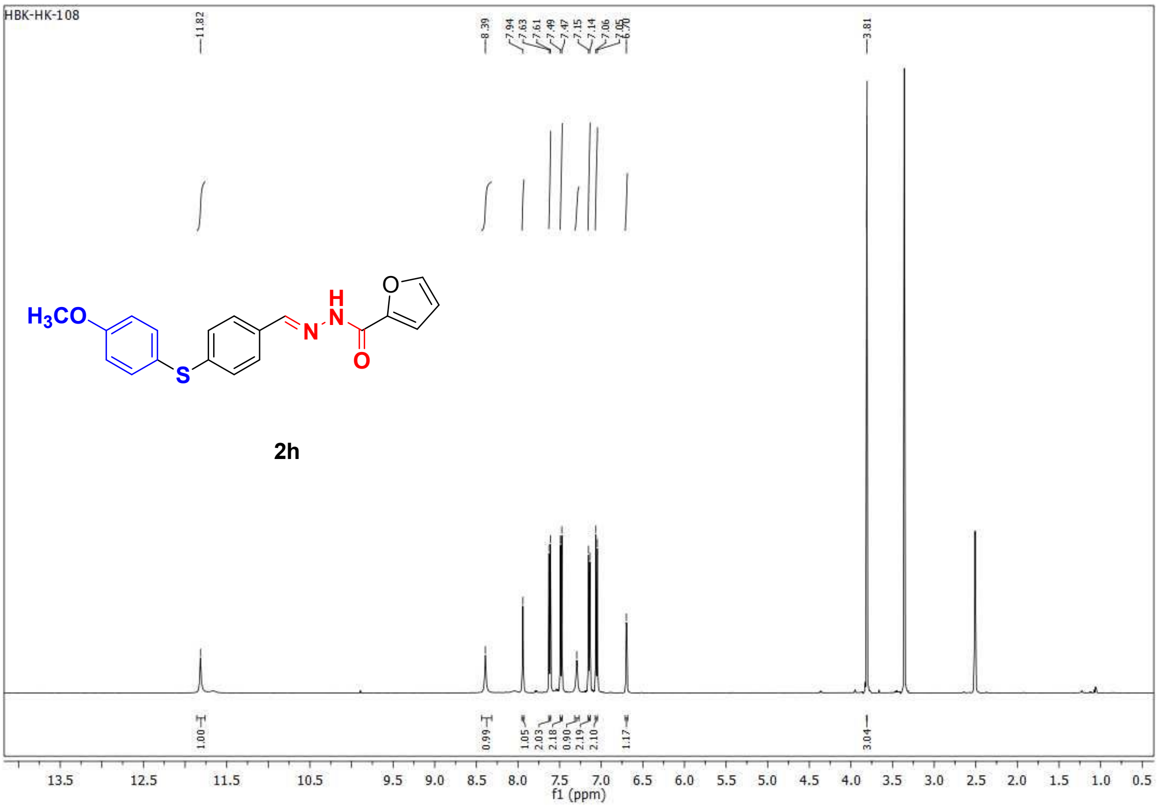

Figure S22. ^{1}H-NMR spectra of compound **2h**.

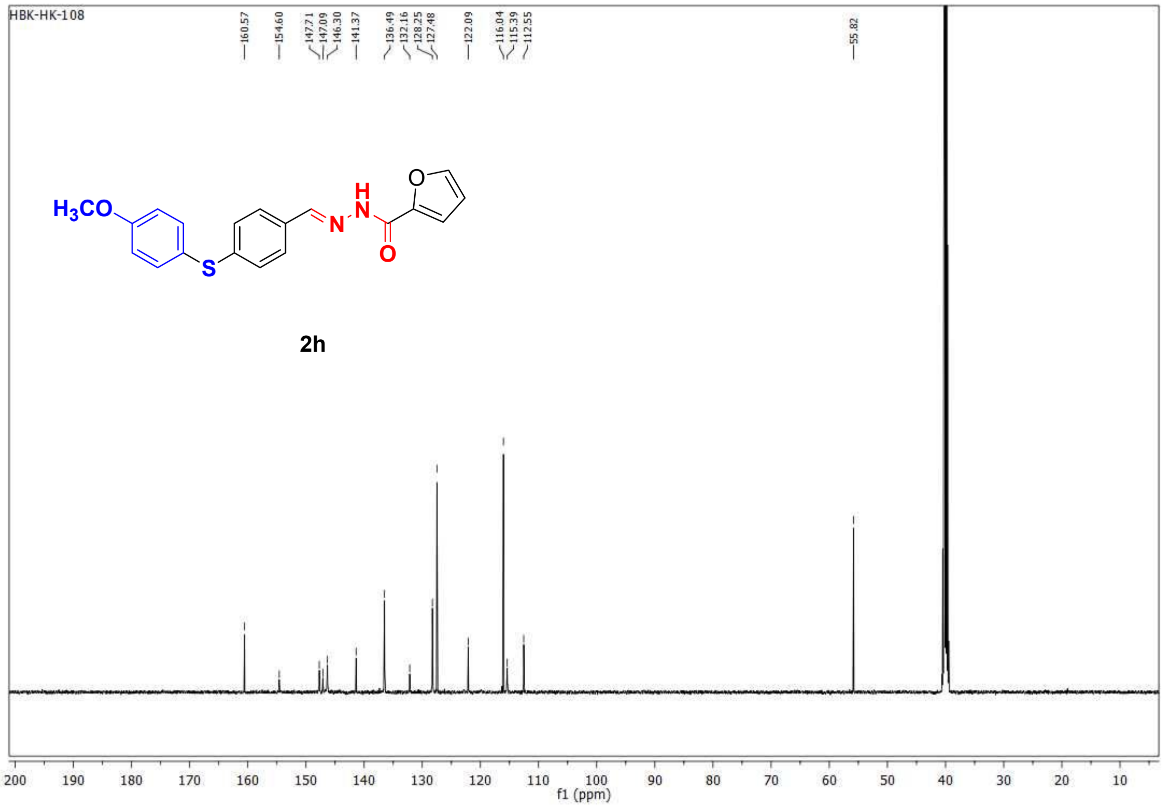

Figure S23. ^{13}C-NMR spectra of compound **2h**.

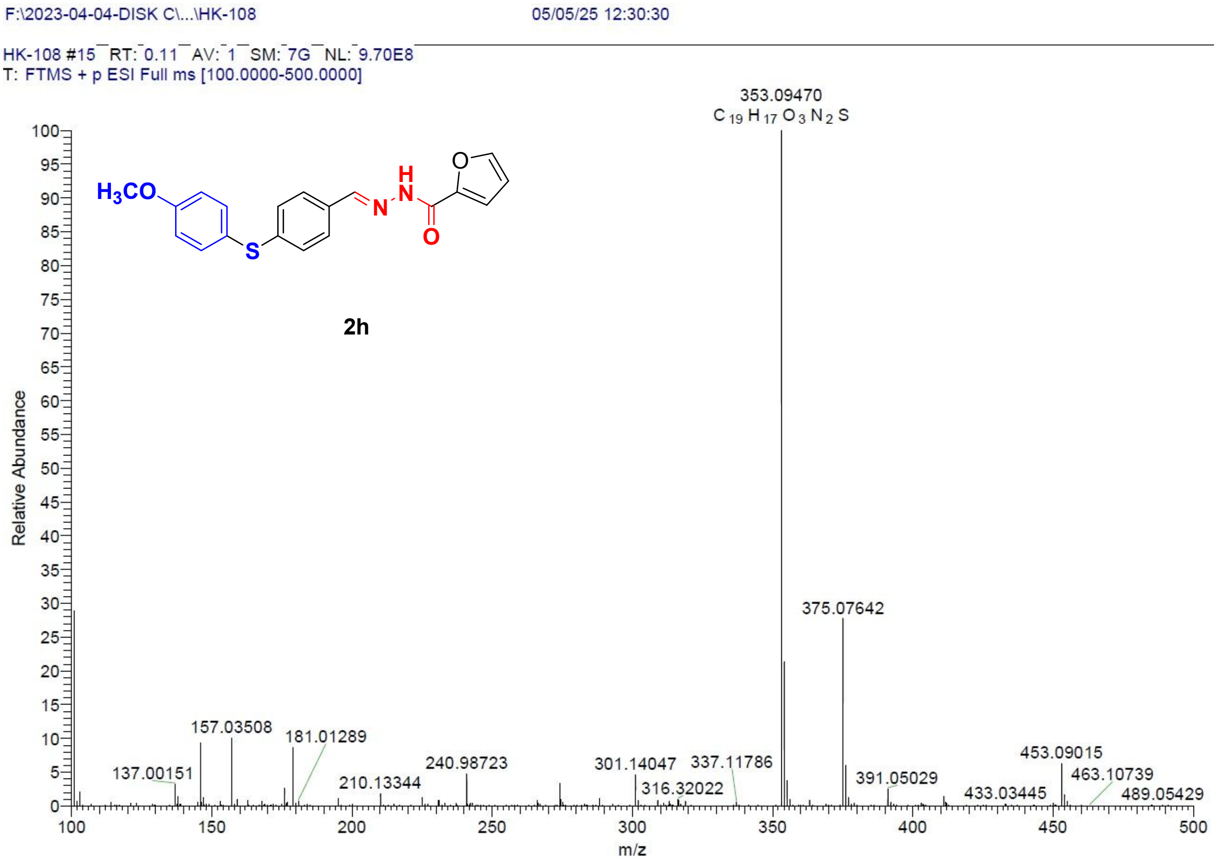

Figure S24. HRMS spectra of compound **2h**.

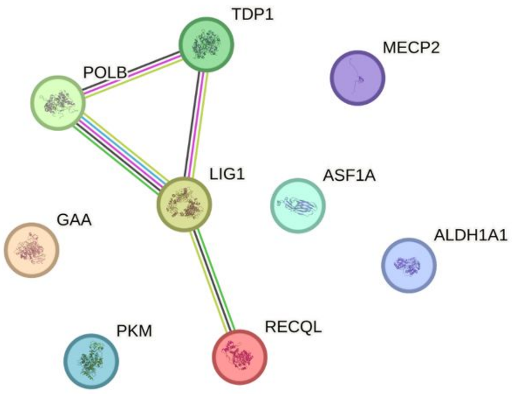

Node1	Node2	Node1 accession	Node2 accession	Score
POLB	LIG1	ENSP00000265421	ENSP00000263274	0,996
LIG1	POLB	ENSP00000263274	ENSP00000265421	0,996
TDP1	POLB	ENSP00000337353	ENSP00000265421	0,963
POLB	TDP1	ENSP00000265421	ENSP00000337353	0,963
TDP1	LIG1	ENSP00000337353	ENSP00000263274	0,664
LIG1	TDP1	ENSP00000263274	ENSP00000337353	0,664
RECQL	LIG1	ENSP00000416739	ENSP00000263274	0.572
LIG1	RECQL	ENSP00000263274	ENSP00000416739	0.572

Figure S25. The protein-protein interaction networks among compound **2c**'s targets.

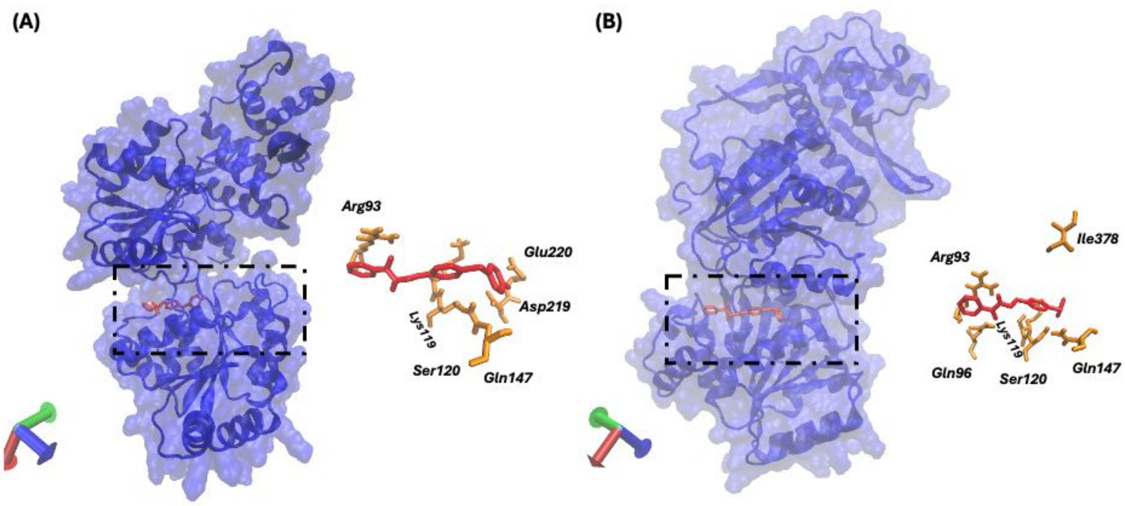

Figure S26. The docking results of compound **2c** to RecQ1 (A) RecQ1 (pdb: 2V1X -chain A) (B) RecQ1 (pdb: 2V1X -chain B).

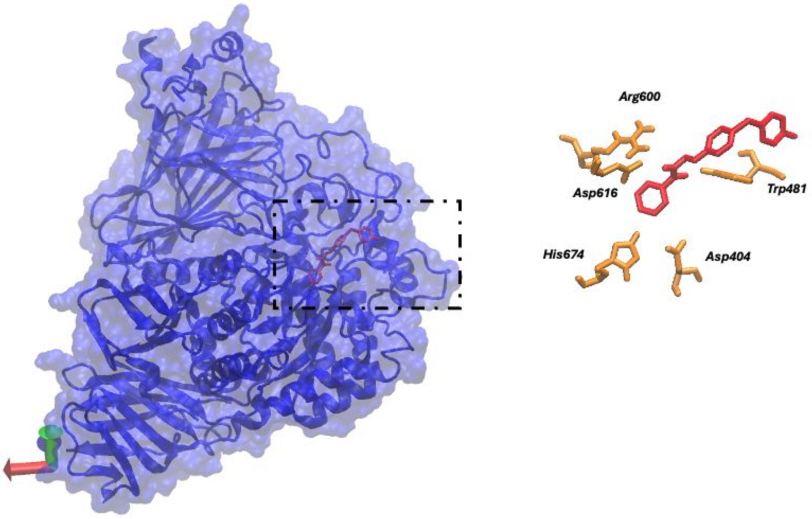

Figure S27. The docking results of compound **2c** to GAA (pdb ID: 5KZX).

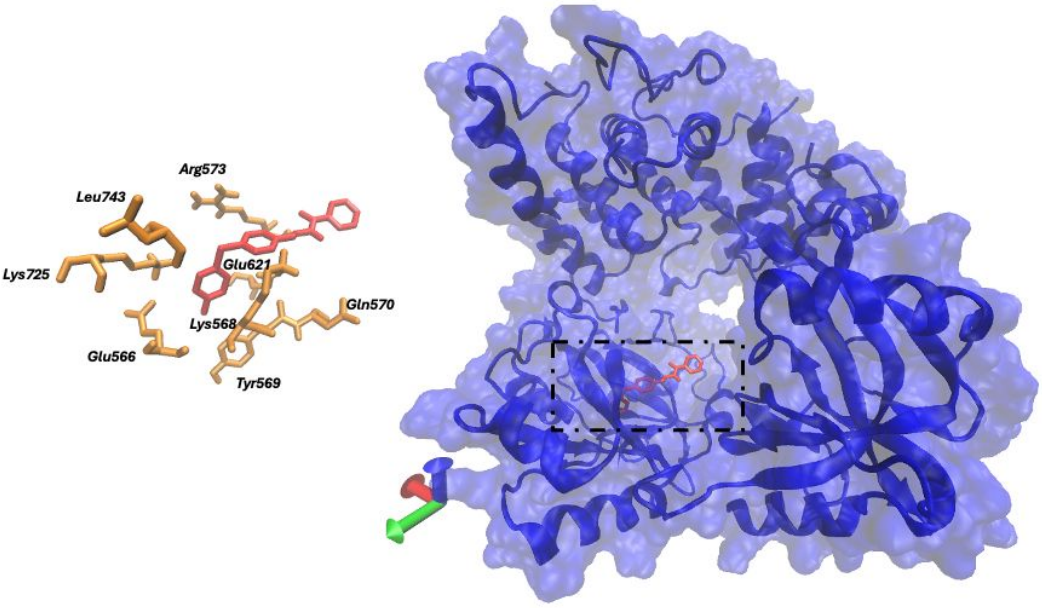

Figure S28. The docking results of compound **2c** to LIG1 (pdb ID: 1X9N).

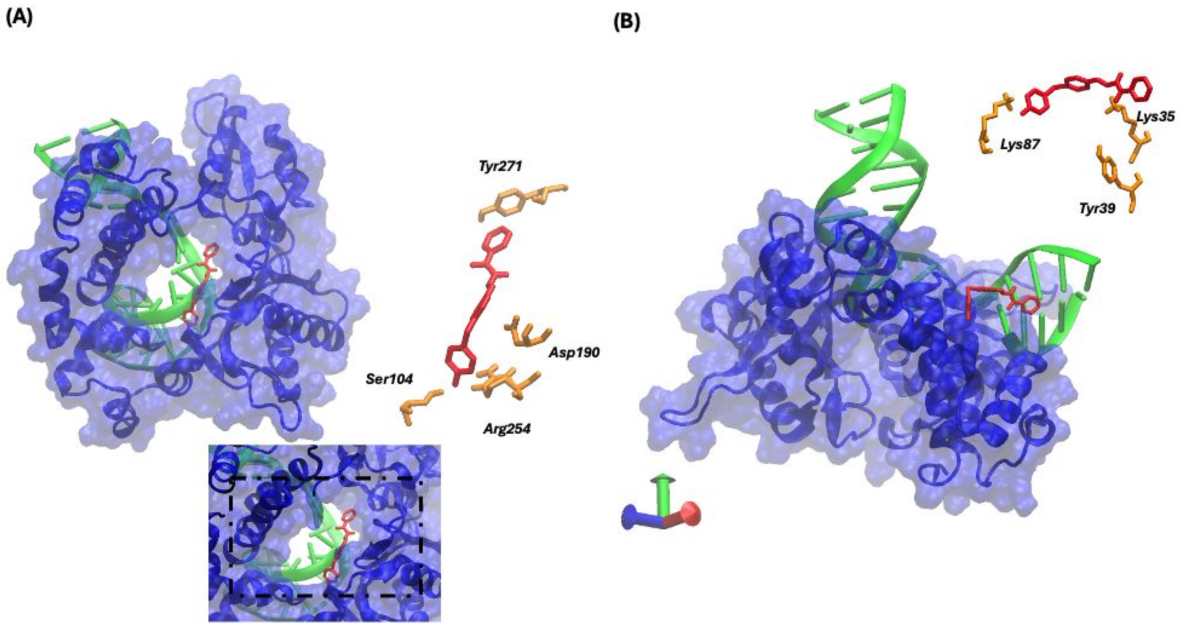

Figure S29. The docking results of compound **2c** to POLB (pdb ID: 1BPX) (A) active site; (B) 5′-deoxyribose phosphate (5′-dRP) binding site.

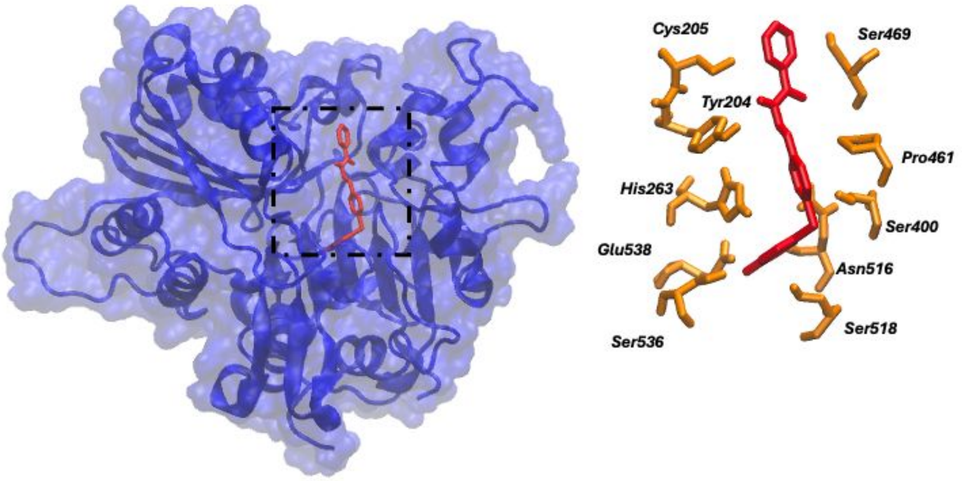

Figure S30. The docking results of compound **2c** to TDP1 (pdb ID: 7UFZ).

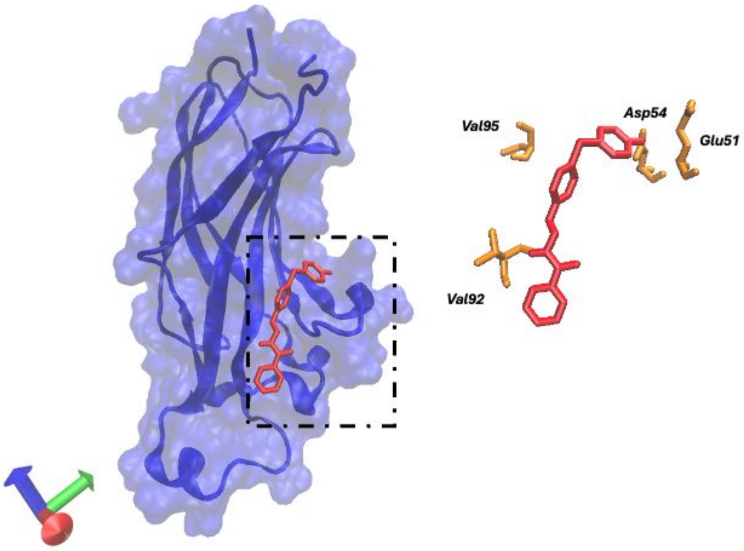

Figure S31. The docking results of compound **2c** to ASF1A (pdb ID: 6F0F).

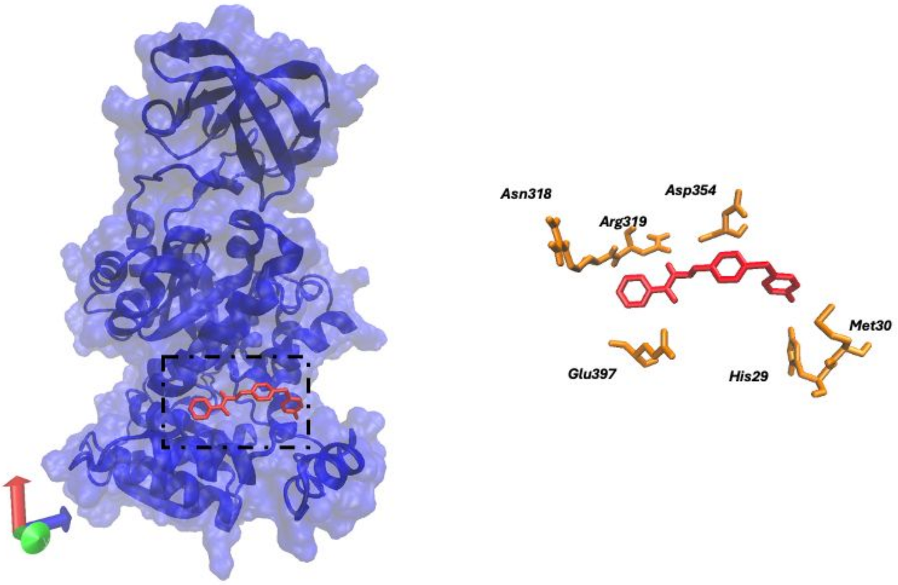

Figure S32. The docking results of compound **2c** to PKM2 (pdb ID: 4G1N).

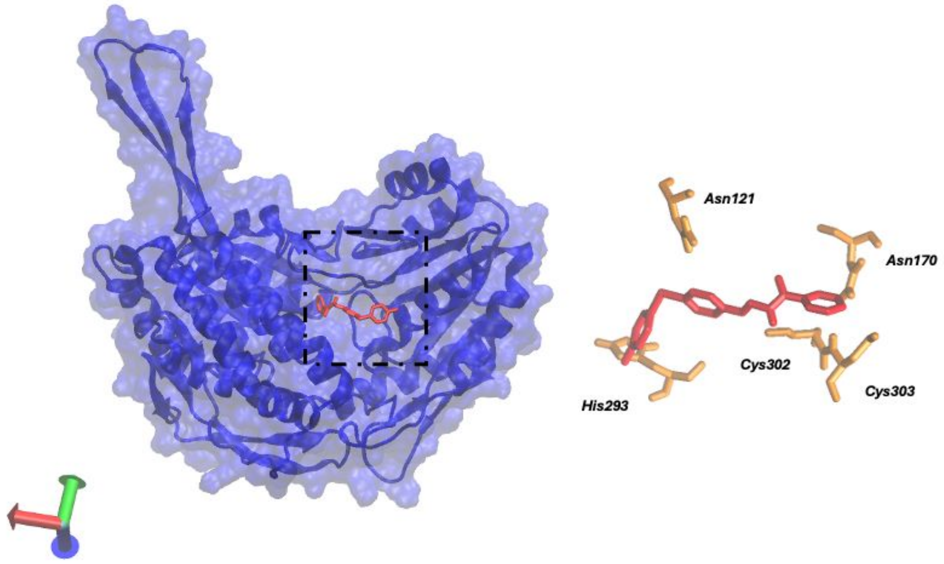

Figure S33. Optimum docking position and dock score of compound **2c** and ALDH1A1 (pdb ID: 4WJ9).

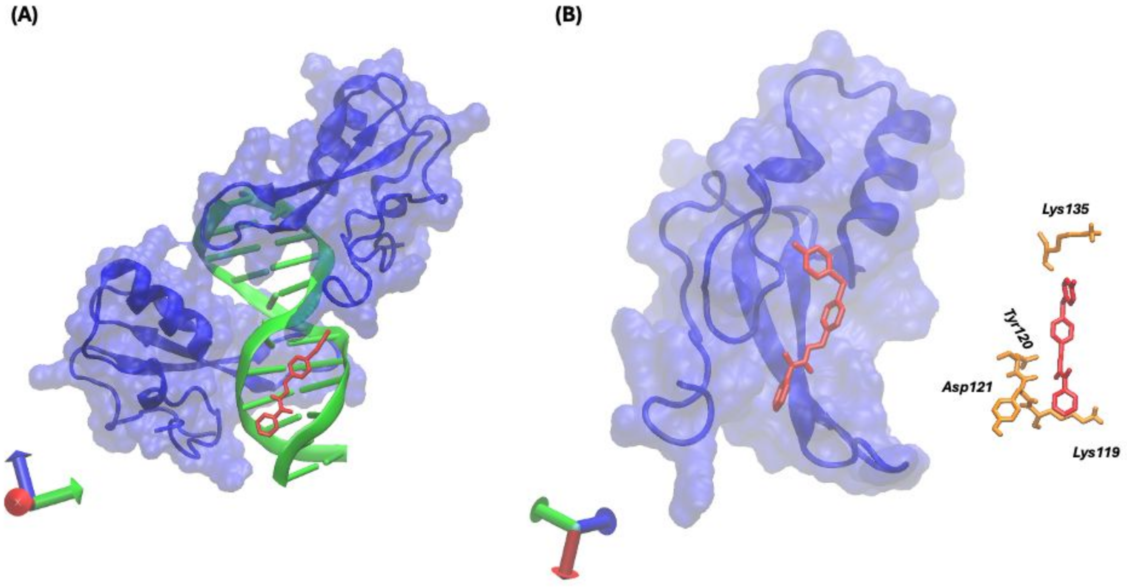

Figure S34. The docking results of compound **2c** to MeCP2 (PDB ID: 6OGJ) (A) DNA-bound MeCP2 and (B) Only MeCP2.

Table S1. Details of the docking score and pose information for compound **2c**.

Protein	PDB ID	Docking Pose	Docking Score (kcal /mole)	RMSD from the best mode u.b.
RECQ1-ChainA	2V1X	1	-7.6	0.0
		2	-7.4	1.696
		3	-6.9	3.135
		4	-6.8	10.364
		5	-6.7	10.714
		6	-6.1	10.918
		7	-6.0	3.703
		8	-5.9	3.250
		9	-5.8	2.519
RECQ1-Cha B	2V1X	1	-7.9	0.0
		2	-7.9	10.361
		3	-7.9	10.404
		4	-7.6	10.233
		5	-7.6	10.498
		6	-7.6	10.538
		7	-7.4	2.823
		8	-7.3	4.888
		9	-7.2	10.763
GAA	5KZX	1	-7.4	0.0
		2	-7.1	10.118
		3	-6.6	9.433
		4	-6.3	10.423
		5	-6.3	10.609
		6	-6.1	11.653
		7	-5.9	8.978
		8	-5.7	7.729
		9	-5.5	9.371
LIG1	1X9N	1	-8.6	0.0
		2	-8.4	1.859
		3	-8.4	2.247
		4	-8.2	10.668
		5	-8.2	2.097
		6	-8.1	6.714
		7	-8	2.229
		8	-7.8	10.704
		9	-7.7	7.279
POLB	1BPX	1	-7.1	0.0
		2	-6.9	1.794
		3	-6.9	10.658
		4	-6.8	6.967
		5	-6.8	10.932
		6	-6.8	10.771
		7	-6.7	1.537
		8	-6.3	3.181
		9	-6.2	11.105
POLB (5′-dRP)	1BPX	1	-4	0.0
		2	-3.2	10.298
		3	-1.5	9.854
		4	-1	10.355
TDP1	7UFZ	1	-6.1	0.0
		2	-5.6	10.789

		3	-5.4	10.525
		4	-4.5	2.101
		5	-4.1	10.710
		6	-4	10.424
		7	-3.9	10.249
		8	-3.6	2.489
		9	-3.2	10.318
ASF1A	6F0F	1	-6	0.0
		2	-6	10.334
		3	-6	10.442
		4	-5.8	10.372
		5	-5.6	2.066
		6	-5.5	10.360
		7	-5.4	2.573
		8	-5.2	11.150
		9	-5.2	2.711
PKM2	4G1N	1	-7.4	0.0
		2	-7.4	10.570
		3	-7.2	3.221
		4	-7	9.882
		5	-6.9	10.070
		6	-6.9	9.653
		7	-6.7	10.742
		8	-6.5	10.682
		9	-6.3	10.942
ALDH1A1	4WJ9	1	-8.3	0.0
		2	-8.1	11.006
		3	-8.1	10.555
		4	-8.1	2.631
		5	-7.9	10.961
		6	-7.8	2.222
		7	-7.4	2.162
		8	-7	10.862
		9	-6.6	10.890
MeCP2 w/ DNA complex	6OGJ	1	-8.4	0.0
		2	-8.3	10.844
		3	-8.3	5.920
		4	-8.1	22.485
		5	-8.1	10.710
		6	-8	10.654
		7	-7.9	3.502
		8	-7.8	10.726
		9	-7.8	13.710
MeCP2 w/o DNA	6OGJ	1	-6.7	0.0
		2	-6.6	24.372
		3	-6.3	18.653
		4	-6.2	23.585
		5	-6.1	12.055
		6	-6.1	25.050
		7	-6	21.955
		8	-6	31.907
		9	-5.9	26.187

References

[1] T. Yildiz, H.B. Küçük, An organocatalytic method for the synthesis of some novel xanthene derivatives by the intramolecular Friedel-Crafts reaction, RSC Adv. 7 (2017) 16644–16649. https://doi.org/10.1039/c6ra27094h.

[2] V.T. Angelova, T. Pencheva, N. Vassilev, R. Simeonova, G. Momekov, V. Valcheva, New indole and indazole derivatives as potential antimycobacterial agents, Med. Chem. Res. 2019 284. 28 (2019) 485–497. https://doi.org/10.1007/S00044-019-02293-W.
